# Jiuwei Xiaozhi Decoction Alleviates High‐Fat Diet‐Induced MASLD by Suppressing Hepatic SREBP2‐Driven Cholesterogenesis and Restoring PPARα‐Mediated Fatty Acid Oxidation

**DOI:** 10.1111/cbdd.70371

**Published:** 2026-07-28

**Authors:** Youcheng Ma, Xinrui Gao, Ji Wang, Lingru Li, Zhen Huang, Fangli Li

**Affiliations:** ^1^ Shenzhen Hospital Beijing University of Chinese Medicine Shenzhen China; ^2^ National Institute of TCM Constitution and Preventive Medicine Beijing University of Chinese Medicine Beijing China

**Keywords:** hepatic lipid metabolism, Jiuwei Xiaozhi decoction, metabolic dysfunction‐associated steatotic liver disease, network pharmacology, PPARα, SREBP2, traditional Chinese medicine

## Abstract

Jiuwei Xiaozhi Decoction (JWXZ) is a nine‐herb traditional Chinese medicine formula clinically used for hepatic steatosis and dyslipidemia, but its chemical basis and pharmacological mechanisms in metabolic dysfunction‐associated steatotic liver disease (MASLD) remain incompletely defined. The chemical profile of JWXZ was characterized by UHPLC‐HRMS/MS. Its therapeutic effects were evaluated in a high‐fat diet (HFD)‐induced mouse model of MASLD, followed by hepatic transcriptomic analysis, network pharmacology, molecular docking, Western blotting, and immunofluorescence validation. UHPLC‐HRMS/MS identified 178 chemical constituents in JWXZ, including phenolic acids, flavonoids, flavonoid glycosides, alkaloids, isoflavones, and coumarins. In HFD‐fed mice, JWXZ attenuated body weight gain, white adipose accumulation, dyslipidemia, hepatic triglyceride and cholesterol accumulation, serum transaminase elevation, and hepatic steatosis, while improving glucose tolerance and partially restoring whole‐body metabolic flexibility. Hepatic transcriptomics identified 124 HFD‐dysregulated genes reversed by high‐dose JWXZ, with prominent enrichment in cholesterol and sterol biosynthesis, endoplasmic reticulum proteostasis, oxidative stress response, and PPARα‐related lipid handling. Integrated transcriptomic and network pharmacology analyses further identified 157 shared targets and 18 overlapping pathways, converging mainly on cholesterol metabolism and PPAR signaling. Protein‐level validation showed that JWXZ suppressed SREBP2 proteolytic activation, reduced nuclear SREBP2 abundance, and downregulated HMGCR, SQLE, and PCSK9, indicating inhibition of the hepatic cholesterogenic program. In parallel, JWXZ restored nuclear PPARα abundance and CPT1A expression, consistent with reactivation of fatty acid oxidation. JWXZ also reduced hepatic EGFR phosphorylation and lowered hepatic and circulating TNF‐α, IL‐6, IL‐1β, and MCP‐1 levels. Molecular docking provided supportive evidence for potential interactions between representative JWXZ constituents and key regulators, including naringin‐HMGCR, chlorogenic acid‐SQLE, rhoifolin‐PPARα, and rhoifolin‐SREBP2. These findings suggest that JWXZ ameliorates HFD‐induced MASLD mainly by rebalancing hepatic lipid metabolism through coordinated suppression of SREBP2‐mediated cholesterogenesis and restoration of PPARα‐mediated fatty acid oxidation, with associated attenuation of EGFR‐linked inflammatory responses.

## Graphical abstract

Studies have found that Jiuwei Xiaozhi Decoction can improve high‐fat diet‐induced fatty liver disease, mainly by regulating the SREBP2 and PPARα pathways to balance hepatic lipid metabolism while reducing inflammatory responses.

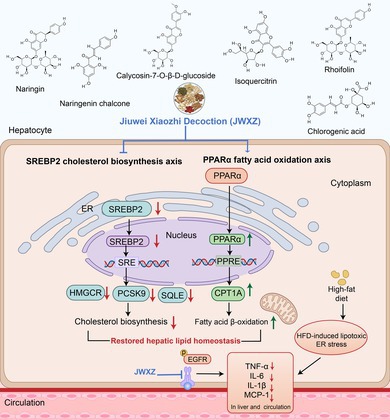

## 1 Introduction

Metabolic dysfunction‐associated steatotic liver disease (MASLD), originally called nonalcoholic fatty liver disease (NAFLD), was recently renamed to better represent its metabolic etiology. It is now a frequently seen global chronic hepatopathy, its prevalence rate is 38% of the adult population, and it is steadily growing (Miao et al. 2024; Zhou et al. 2023). MASLD is a broad spectrum of hepatopathy, encompassing simple hepatic steatosis to metabolic dysfunction‐associated steatohepatitis (MASH), liver fibrosis, cirrhosis, or even hepatocellular carcinoma. As a result, it has become an important factor contributing to end‐stage liver disease as well as liver transplantation on a global scale (Harrison, Rolph, et al. 2024). The MASLD pathogenic mechanism is predetermined by a complicated and closely interconnected system of pathological processes. Among them, the dysregulated hepatic lipid metabolism, especially the disruption of cholesterol and fatty acid homeostasis, is one of the primary upstream factors that facilitate lipotoxicity, endoplasmic reticulum (ER) stress, and chronic lower‐grade inflammation (Carli et al. 2024; Venkatesan et al. 2023). Moreover, there is rising evidence of two important metabolic pathways in disease development and progression: cholesterol biosynthesis modulated via sterol regulatory element‐binding protein 2 (SREBP2) and fatty acid oxidation (FAO) modulated by peroxisome proliferator‐activated receptor alpha (PPARα). The imbalance between these pathways has been identified as one of the key factors that lead to hepatic steatosis and MASLD progression (Barbhuiya et al. 2025; Krahmer et al. 2025).

Although MASLD has a significant global burden, there is a lack of effective pharmacological interventions. Presently, there are only approved drug treatments for MASH that are limited to particular groups of patients. As a thyroid hormone receptor‐β agonist, resmetirom has been licensed for adult patients developing noncirrhotic MASH and moderate‐to‐advanced liver fibrosis (Harrison, Bedossa, et al. 2024), and semaglutide has recently been licensed in such adult patients (Sanyal et al. 2025). Nevertheless, they are only recommended for specific groups of patients and not to the entire population of MASLD. Furthermore, important challenges related to long‐term efficacy, clinical outcomes, cost‐effectiveness, tolerability, and therapeutic performance across the heterogeneous spectrum of MASLD remain unresolved (European Association for the Study of the Liver et al. 2024). Since MASLD represents a heterogeneous disease involving multiple factors, accompanied by complicated interactions between genetic predisposition, insulin resistance, dyslipidemia, and dietary factors, it is unlikely that a single molecular target will be effective in treating the entire range of disease pathogenesis (Sheikh et al. 2025). As a result, multi‐target therapeutic interventions that can simultaneously control hepatic lipid metabolism, energy homeostasis, and inflammatory reactions have become a promising option in the management of MASLD.

Multi‐herb formulations and traditional Chinese medicine (TCM) are extensively employed for treating metabolic disorders and have gained extensive clinical experience in this area. There is growing pharmacological evidence that TCM has positive effects in MASLD by coordinating lipid metabolism, energy homeostasis, gut microbiota composition, and inflammatory signaling pathways (Tao et al. 2024). Jiuwei Xiaozhi Decoction (JWXZ) is a traditional nine‐herb preparation that has been used clinically to treat hepatic steatosis and dyslipidemia (Li, Ni, et al. 2015; Li, Xu, et al. 2015). It consists of *Astragali Radix*, *Crataegi Fructus*, *Coicis Semen*, *Atractylodis Rhizoma*, *Mori Folium*, *Citri Grandis Exocarpium*, *Poria*, *Myristicae Semen*, and *Raphani Semen*. JWXZ is based on two classical TCM prescriptions, Pingwei San and Baohe Wan, which are traditionally used to treat spleen‐stomach dysfunction and food stagnation. In light of contemporary pathophysiology, these classical ideas can be generally related to the disruption of digestive and metabolic activity, and the disruption of lipid and glucose homeostasis related to chronic overnutrition. It has been previously shown that prescriptions based on Pingwei San can reduce high‐fat diet (HFD)‐mediated metabolic dysfunction and intestinal inflammation (Fan et al. 2023), and Baohe Wan can ameliorate high‐fat and high‐protein diet‐mediated gut microbial dysbiosis (Guo et al. 2025). On the individual herbal constituents level, bioactive constituents of Astragalus have been documented to enhance hepatic lipid metabolism and insulin resistance (Zheng et al. 2024). Likewise, hawthorn flavonoids reduce hepatic steatosis via AMP‐activated protein kinase (AMPK)‐related pathways (Li, Ni, et al. 2015; Li, Xu, et al. 2015), and Coicis Semen, Mori Folium, Citri Grandis Exocarpium, and Poria have been shown to have lipid‐lowering and hepatoprotective effects on experimental dyslipidemia and hepatic steatosis models (Ann et al. 2015; He et al. 2022; Jiang et al. 2024; Lee et al. 2020). Collectively, such findings provide a strong pharmacological foundation for investigating JWXZ as the multi‐component, multi‐target anti‐MASLD treatment. However, those specific bioactive compounds responsible for its therapeutic efficacy, as well as the molecular pathways through which JWXZ regulates hepatic lipid metabolism, remain largely undefined.

In the present study, chemical profiling was conducted in combination with in vivo pharmacological assessment, hepatic transcriptomic analysis, molecular docking, and network pharmacology for systematically elucidating the bioactive basis and therapeutic mechanisms of JWXZ in MASLD. JWXZ was comprehensively analyzed for its chemical components by utilizing ultra‐high‐performance liquid chromatography coupled with high‐resolution tandem mass spectrometry (UHPLC‐HRMS/MS). Its therapeutic efficacy was subsequently assessed with an HFD‐induced murine model of MASLD through the evaluation of body composition, glucose and lipid metabolism, whole‐body energy expenditure, and hepatic histopathological alterations. To clarify the associated molecular mechanisms, we performed integrated transcriptomic and network pharmacology analyses. Then, core target proteins were subjected to molecular docking studies as well as in vivo verification. Overall, this study aimed to establish a comprehensive chemical and mechanistic framework for understanding the protection of JWXZ on HFD‐related MASLD and to provide a certain basis for developing it as the multi‐target treatment against metabolic hepatopathy.

## 2 Materials and Methods

### 2.1 Preparation of JWXZ Decoction

JWXZ decoction was prepared from Astragali Radix (20 g), Myristicae Semen (10 g), Crataegi Fructus (20 g), Coicis Semen (20 g), Atractylodis Rhizoma (20 g), Mori Folium (30 g), Citri Grandis Exocarpium (10 g), Poria (20 g), and Raphani Semen (10 g), with a total crude herb weight of 160 g. These herbs were immersed within 2 L of purified water for 1 h and then decocted for 1 h. The remaining herbal residue (marc) was subsequently re‐decocted for 30 min with an additional volume of water sufficient to fully immerse the material. The filtrates obtained from both decoctions were combined and clarified to remove insoluble particulates, followed by concentration at reduced pressure and freeze‐drying to yield a dry extract, which was used for subsequent chemical profiling and experimental studies.

### 2.2 UHPLC‐HRMS/MS Profiling and Compound Annotation

For chemical analysis, 200 mg of JWXZ powder was extracted using 10 mL of 50% methanol–water solution (v/v, 50:50) by ultrasonication for 30 min. Following 5 min of centrifugation (14,000 *g*), supernatants were gathered and filtered with the 0.22‐μm membrane prior to analysis. A blank control sample was prepared simultaneously using the same extraction procedure. The ACQUITY UPLC HSS T3 column (Waters, 1.8 μm, 2.1 × 100 mm) was adopted for chromatographic separation, which was kept under 35°C, with an injection volume and flow rate of 10 μL and 0.3 mL/min respectively. The mobile phase contained 0.1% formic acid in water (solvent A) alongside 0.1% formic acid within acetonitrile (solvent B). The program below was adopted for elution: 0–10 min, 0%–30% B; 10–25 min, 30%–40% B; 25–30 min, 40%–50% B; 30–40 min, 50%–70% B; 40–45 min, 70%–100% B; 45–60 min, 100% B; 60–60.5 min, 100%–0% B; and 60.5–70 min, 0% B. Mass spectrometric analysis was performed using a Q Exactive Plus Orbitrap mass spectrometer (Thermo Fisher Scientific) equipped with an electrospray ionization (ESI) source. Data were acquired in both positive and negative ion modes using the Full MS/dd‐MS^2^ acquisition mode over an *m*/*z* scan range of 100–1200. The resolutions for MS1 and MS2 scans were set to 70,000 and 17,500, respectively. Instrument parameters were as follows: sheath gas flow rate, 40 arbitrary units; auxiliary gas flow rate, 15 arbitrary units; capillary temperature, 320°C; auxiliary gas heater temperature, 350°C; spray voltage, 3.2 kV; automatic gain control (AGC) target, 1 × 10^6^; and TopN, 5. For MS/MS fragmentation, stepped normalized collision energies were set to 30, 40, and 50. Processing of raw LC–MS data was accomplished with Compound Discoverer 3.2 software (Thermo Fisher Scientific). Putative compounds were identified by matching exact mass measurements and MS/MS fragmentation spectra against the mzCloud database and an in‐house mzVault spectral library, using a mass tolerance threshold of 5 ppm. Candidate compounds were further screened using an mzVault matching score greater than 70, followed by manual verification and removal of redundant annotations to improve annotation reliability.

### 2.3 Transcriptome Sequencing and Bioinformatics Analysis

#### 2.3.1 Liver Transcriptomic Analysis

Liver transcriptomic profiling was carried out following previous description after mild modifications (Zou et al. 2026). TRIzol reagent was employed for isolating total liver tissue RNA, and RNA quantity was evaluated by the NanoDrop spectrophotometer, while RNA integrity by the Agilent 2100 Bioanalyzer. Sequencing libraries were subsequently prepared for paired‐end sequencing using the Illumina NovaSeq 6000 platform. Additionally, quality control and filtering were completed to obtain clean reads, and HISAT2 was later used to align them to the reference genome. Moreover, gene expression was expressed as RSEM, and DESeq2 package was adopted for conducting differential analysis. In addition, differentially expressed genes (DEGs) were identified in line with the |log_2_FC| ≥ 1 and adjusted *p*‐value < 0.05 thresholds.

To identify genes potentially related to the therapeutic effects of JWXZ, we focused on genes exhibiting a reversed expression pattern. Specifically, these genes were significantly dysregulated in the HFD versus NC comparison and displayed opposite regulatory trends in the JWXZ‐H versus HFD comparison. Genes meeting these criteria were designated as reversed genes and were subsequently subjected to downstream analyses. To visualize the data, the top 40 reversed genes were chosen based on the magnitude of their changes in expression and statistical significance. The biological meaning of the identified reversed genes was investigated by conducting functional enrichment analyses. By using the DAVID database, GO and KEGG analyses were conducted with *Mus musculus* as the reference organism. Enriched terms and pathways meeting the predefined significance criteria were retained for subsequent interpretation.

#### 2.3.2 Network Pharmacology Analysis

##### 2.3.2.1 Prediction of Potential Targets for JWXZ and MASLD

Secondary metabolites detected through UHPLC‐HRMS/MS were selected for subsequent target prediction. To minimize background interference with primary metabolites, we eliminated ubiquitous small molecules, including sugars, nucleotides, fatty acids, and amino acids, and retained secondary metabolites whose structures were characterized (Cho et al. 2025). Compounds meeting the following criteria were selected for target prediction: mass error < 5 ppm, annotation score > 70, and relative peak area > 0.5%. Ultimately, 15 representative bioactive compounds were obtained.

A combined analysis integrating database‐predicted targets with transcriptome‐derived DEGs was done to explain the potential mechanisms of JWXZ, with minor modifications to previous description (Zhao et al. 2025). The SwissTargetPrediction platform (http://www.swisstargetprediction.ch/) was employed for predicting the selected compounds' potential targets. In parallel, DEGs identified from transcriptomic analysis were also included as candidate targets of JWXZ. All target proteins were standardized by mapping gene names to UniProt database (https://www.uniprot.org/), and the species were set as *Homo sapiens* , and duplicate entries were eliminated when constructing a unified JWXZ target database.

Disease‐associated targets were obtained based on the GeneCards (https://www.genecards.org/), the Comparative Toxicogenomics Database (CTD, https://ctdbase.org/), as well as Online Mendelian Inheritance in Man (OMIM) (https://www.omim.org/) databases using the keywords “MASLD,” “NAFLD,” and “metabolic dysfunction‐associated fatty liver disease.” Targets obtained in the above three databases were integrated, while redundant or invalid entries were eliminated for generating an integrated disease target dataset.

The associations of JWXZ targets with disease targets were analyzed, and a Venn diagram was generated utilizing the Bioinformatics platform (https://www.bioinformatics.com.cn/) for visualizing the overlapping targets.

##### 2.3.2.2 Establishment of Protein–Protein Interaction (PPI) Networks

PPI analysis of the overlapping targets between JWXZ and MASLD was done employing the STRING database (https://string‐db.org/), and isolated nodes were excluded from the network construction.

##### 2.3.2.3 GO and KEGG Analysis

To explain potential biological pathways related to the efficacy of JWXZ in MASLD, KEGG as well as GO analysis was carried out with DAVID database (https://david.ncifcrf.gov/), with *Homo sapiens* being set to reference species. Terms satisfying an adjusted *p*‐value < 0.05 showed significant enrichment. Metascape (https://metascape.org/) was further employed to conduct complementary enrichment. Moreover, visualization of enrichment findings was completed utilizing the Bioinformatics online platform (https://www.bioinformatics.com.cn/).

### 2.4 Animal Experiment Design

Five‐week‐old male C57BL/6N mice (19–21 g) were provided by Beijing Vital River Laboratory Animal Technology Co. Ltd. (Beijing, China; production license No. SCXK (Beijing) 2021‐0006). Mice were housed under SPF conditions in Beijing University of Chinese Medicine (45%–65% relative humidity, 20°C–24°C) with a 12‐h/12‐h light/dark cycle (lights on 08:00–20:00). The experimental protocols were approved by the Institutional Animal Care and Use Committee of Beijing University of Chinese Medicine (Approval No. BUCM20250729‐001).

A total of 32 SPF male C57BL/6N mice were used. In accordance with a previous report (Sun, Jia, et al. 2025), a MASLD model was induced using an HFD. Table S1 presents the experimental diet composition. In total, 32 animals were randomized into four groups (*n* = 8 per group): normal control (NC; standard chow), HFD model, low‐dose JWXZ (JWXZ‐L, 0.512 g kg^−1^ day^−1^), and high‐dose JWXZ (JWXZ‐H, 1.023 g kg^−1^ day^−1^). The HFD, JWXZ‐L, and JWXZ‐H groups received an HFD throughout the 12‐week experimental period, whereas NC mice received standard chow. JWXZ was given orally every day for a 12‐week period.

At the end of the study, animals underwent a 12‐h fasting period, and water was provided ad libitum. Blood was collected, and plasma was obtained by 5 min of centrifugation (1500 *g*, 4°C). Liver and adipose samples were immediately excised, which were either snap‐frozen within liquid nitrogen for subsequent molecular analyses or immersed within 10% formalin for histological evaluation. Isoflurane anesthesia was used to minimize procedural discomfort, and animals were euthanized via carbon dioxide inhalation.

### 2.5 Oral Glucose Tolerance Test (OGTT)

Following overnight fasting, animals subjected to an OGTT. The blood glucose levels were measured at 0, 15, 30, 60, 90, and 120 min after oral glucose administration. The trapezoidal method of calculating the area under the curve (AUC) was applied in GraphPad Prism 10.0.

### 2.6 Indirect Calorimetry

By using a TSE indirect calorimetry system (TSE Systems, Bad Homburg, Germany), the whole‐body energy metabolism was determined in the NC, HFD, and JWXZ‐H groups. Mice were placed in metabolic chambers with food and water ad libitum and placed in the 12‐h light/dark cycle. Following adaptive feeding, the respiratory exchange ratio (RER) and heat production were measured continuously during a 24 h period, and the time‐course data obtained were analyzed later.

### 2.7 Biochemical Assays

Serum levels of alanine aminotransferase (ALT), aspartate aminotransferase (AST), high‐density lipoprotein cholesterol (HDL‐C), low‐density lipoprotein cholesterol (LDL‐C), glucose (GLU), total cholesterol (TC), and triglycerides (TG) were determined by commercial assay kits (Nanjing Jiancheng Bioengineering Institute, Nanjing, China). TC and TG contents within the liver were measured using the corresponding kits. The BCA protein assay kit (Beijing Solarbio Science & Technology Co. Ltd., Beijing, China) was adopted for quantifying total protein concentrations within liver homogenates. Fasting serum insulin was quantified utilizing a mouse insulin ELISA kit (Beijing LABLEAD Inc., Beijing, China). TNF‐α, IL‐6, MCP‐1, and IL‐1β contents in serum and liver were determined with commercial ELISA kits (Beijing LABLEAD Inc., Beijing, China) according to manufacturers' instructions.

### 2.8 Tissue Collection and Histology

Both liver and adipose samples were collected from euthanized mice according to a previously described protocol (Kong et al. 2026). Deposits of adipose were brown adipose tissue (BAT) and white adipose tissue (WAT). The WAT contains inguinal white adipose tissue (iWAT) and epididymal white adipose tissue (eWAT). Wet weights of each adipose tissue and the liver were recorded. Total white adipose tissue (total WAT) was determined as the total of eWAT and iWAT. Relative tissue mass was calculated as tissue weight divided by body weight (mg/g). The liver tissues were cut into two portions, with one part snap‐frozen and kept at −80°C to be subjected to further analyses, and the other part fixed in formalin to be examined under a microscope. Tissue sections later received paraffin embedding and hematoxylin and eosin staining (H&E; C0105S, Beyotime, China). Pathomorphological alterations such as adipocyte hypertrophy or hepatic steatosis were assessed under the light microscope with a 40× objective lens.

### 2.9 Western Blotting Analysis

Total liver proteins were extracted from frozen liver tissues using RIPA lysis buffer supplemented with protease and phosphatase inhibitors. For subcellular protein analysis, nuclear and cytoplasmic proteins were extracted from liver tissues using NE‐PER Nuclear and Cytoplasmic Extraction Reagents (Thermo Fisher Scientific, Cat. No. 78833) according to the manufacturer's instructions. Briefly, liver tissues were homogenized on ice in cytoplasmic extraction buffer containing protease and phosphatase inhibitors. After centrifugation at 4°C, the supernatant was collected as the cytoplasmic fraction. The remaining nuclear pellet was washed and further lysed in nuclear extraction buffer, followed by centrifugation to obtain the nuclear protein fraction.

Protein concentrations were determined using a BCA protein assay kit. Equal amounts of protein were separated by SDS‐PAGE and transferred onto PVDF membranes. The membranes were blocked with 5% non‐fat milk or 5% BSA, depending on the primary antibody, and then incubated overnight at 4°C with primary antibodies against SREBP2, PPARα, HMGCR, SQLE, PCSK9, CPT1A, EGFR, p‐EGFR, PI3K, p‐PI3K, AKT, p‐AKT, Lamin B1, and GAPDH. After washing, the membranes were incubated with HRP‐conjugated goat anti‐rabbit IgG or goat anti‐mouse IgG secondary antibodies at room temperature. Protein bands were visualized using an enhanced chemiluminescence detection system and quantified with ImageJ software.

For normalization, phosphorylated proteins were normalized to their corresponding total protein levels. Total liver proteins were normalized to GAPDH. For nuclear and cytoplasmic fractionation analysis, N‐SREBP2 and N‐PPARα were normalized to Lamin B1, whereas C‐PPARα was normalized to GAPDH. Detailed information on all primary and secondary antibodies is provided in Table S4.

### 2.10 Immunofluorescence Staining

Liver samples were fixed in 4% paraformaldehyde, embedded in paraffin, and sectioned at 4–5 μm thickness. After deparaffinization and rehydration, antigen retrieval was performed using citrate buffer. The sections were permeabilized with Triton X‐100 and blocked with 5% BSA to reduce nonspecific binding. Subsequently, the sections were incubated overnight at 4°C with primary antibodies against SREBP2 and PPARα. After washing, the sections were incubated with CoraLite594‐conjugated Goat Anti‐Rabbit IgG(H + L) secondary antibody (Proteintech, SA00013‐4, 1:500) or CoraLite488‐conjugated Goat Anti‐Mouse IgG(H + L) secondary antibody (Proteintech, SA00013‐1, 1:500) in the dark at room temperature. Nuclei were counterstained with DAPI.

Fluorescence images were captured using a fluorescence microscope. The subcellular distribution of SREBP2 and PPARα was assessed based on the overlap between target protein fluorescence and DAPI‐positive nuclei. Fluorescence intensity was quantified using ImageJ software. Detailed information on the primary and secondary antibodies used for immunofluorescence staining is listed in Table S4.

### 2.11 Molecular Docking Analysis

Through conducting molecular docking, potential associations of representative JWXZ‐derived compounds with core protein targets identified from the integrated analysis were evaluated. The three‐dimensional structures of HMGCR, SQLE, PPARα, EGFR, and SREBP2 were obtained based on the Protein Data Bank (PDB) or, in the absence of an experimental structure, from the AlphaFold Protein Structure Database (*Homo sapiens*). After searching relevant databases, it was ultimately determined that the structures of HMGCR, SQLE, PPARα, and EGFR were derived from the PDB, while the structure of SREBP2 was obtained from AlphaFold Protein Structure Database (Table 2). To prepare protein structures, water molecule removal, hydrogen atom introduction, and energy minimization of ligand structure were completed before docking. Furthermore, AutoDock Vina was employed for docking simulations, with binding affinities reported as docking scores (kcal/mol). Representative docking conformations and ligand‐residue interactions were visualized utilizing Discovery Studio and PyMOL.

### 2.12 Statistical Analysis

GraphPad Prism 10.0 was used for statistical analysis. Data normality was assessed by the Shapiro–Wilk test before analysis and Levene's test was applied to assess the homogeneity of variances across groups. Normally distributed data were analyzed using one‐way analysis of variance (ANOVA) or Student's *t*‐test, while non‐normally distributed data were analyzed using the Kruskal‐Wallis H test or Mann–Whitney Rank Sum test. When ANOVA identified a significant difference, multiple pairwise comparisons were conducted using Student's *t*‐test with Bonferroni correction. *p* < 0.05 was considered indicative of a significant difference. The number of participants included in each analysis is shown in the figure and table legends.

## 3 Results

### 3.1 Chemical Profiling of JWXZ by UHPLC‐HRMS/MS

**FIGURE 1:**
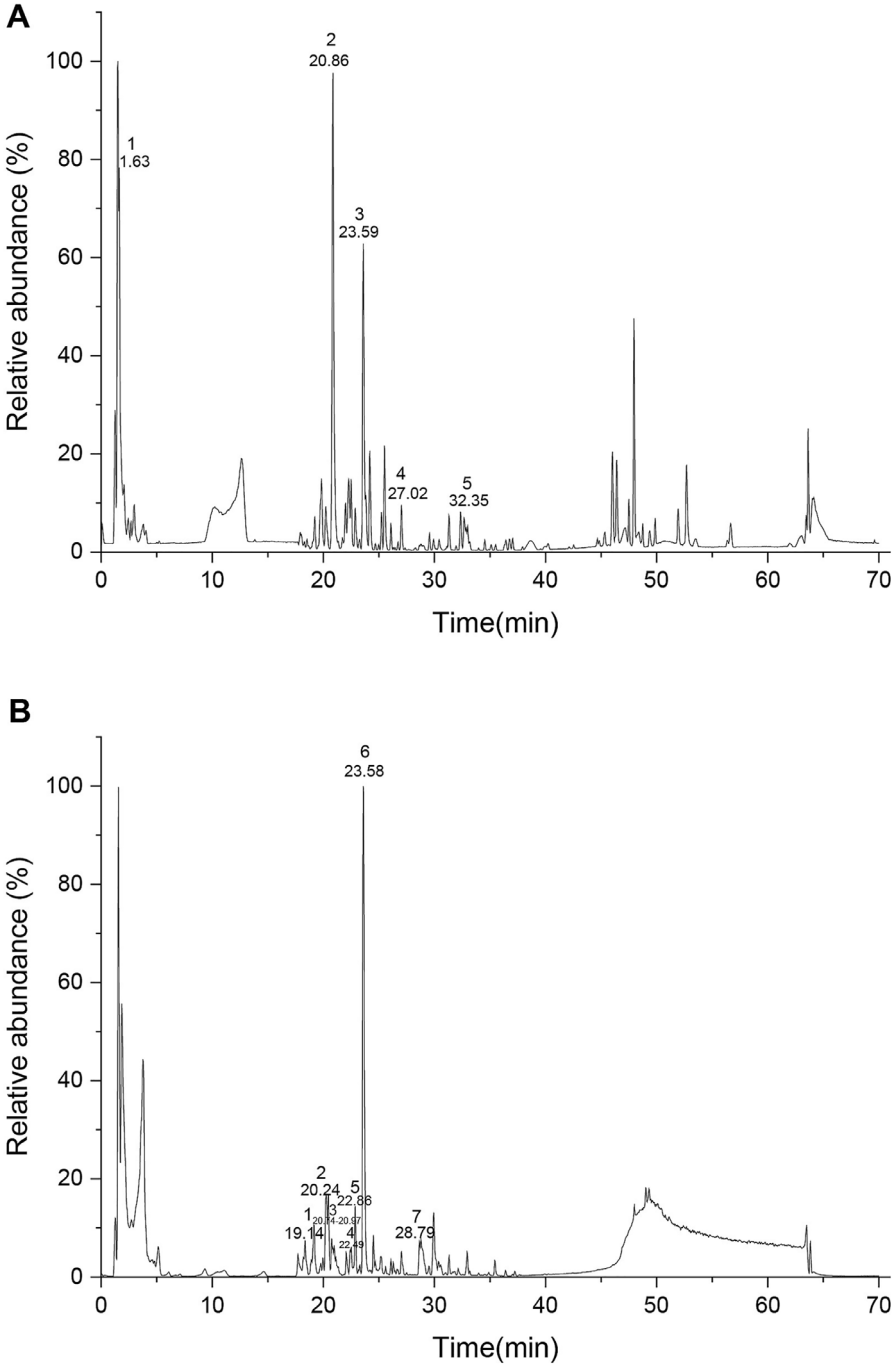
Chemical profiling of JWXZ by UHPLC‐HRMS/MS. (A, B) Total ion chromatograms of JWXZ acquired in positive ion mode (A) and negative ion mode (B). Representative peaks were annotated according to retention time, accurate mass, MS/MS fragmentation, and database matching. Peak numbers correspond to the representative compounds listed in Table 1.

UHPLC‐HRMS/MS analysis was performed to characterize the chemical composition of JWXZ. The total ion chromatograms obtained in negative/positive ion modes displayed multiple well‐resolved peaks, reflecting the complex chemical profile of the formula (Figure 1A,B). A total of 178 chemical components were identified (Table S2), and the major chemical classes are summarized in Table 1, including alkaloids, phenolic acids, flavonoids, and flavonoid glycosides, isoflavones, and coumarins. Representative compounds with relatively high relative abundance included naringin (11.356%), sinapine (8.013%), naringenin chalcone (4.688%), chlorogenic acid (2.302%), Corchorifatty acid F (2.174%), cryptochlorogenic acid (1.349%), trigonelline (1.238%), rhoifolin (1.238%), and isoquercitrin (1.148%). These findings indicate that the structurally diverse secondary metabolites in JWXZ may provide the chemical basis for its pharmacological activities.

**TABLE 1:** Representative secondary metabolites identified in JWXZ by UHPLC‐HRMS/MS.

Peak label	Compound	Chemical class	Molecular formula	*R*_t_ (min)	Mass error (ppm)	Match score	Relative content (%)
A3/B6	Naringin	Flavonoid glycoside	C_27_H_32_O_14_	23.587	−0.83	90.0	11.356
A2/B3	Sinapine	Alkaloid	C_16_H_23_NO_5_	20.850	0.45	89.1	8.013
A3/B6	Naringenin chalcone	Chalcone	C_15_H_12_O_5_	23.582	0.09	90.1	4.688
B2	Chlorogenic acid	Phenolic acid	C_16_H_18_O_9_	20.237	−0.77	93.1	2.302
B7	Corchorifatty acid F	Fatty acid derivative	C_18_H_32_O_5_	28.740	−0.98	88.9	2.174
B1	Cryptochlorogenic acid	Phenolic acid	C_16_H_18_O_9_	19.154	−0.92	92.0	1.349
A1	Trigonelline	Alkaloid	C_7_H_7_NO_2_	1.625	1.51	86.8	1.238
A3/B6	Rhoifolin	Flavonoid glycoside	C_27_H_30_O_14_	23.544	0.30	82.9	1.238
B5	Isoquercitrin	Flavonoid glycoside	C_21_H_20_O_12_	22.861	0.11	93.6	1.148
B4	Calycosin‐7‐O‐β‐D‐glucoside	Isoflavone glycoside	C_22_H_22_O_10_	22.491	0.61	87.2	0.816
B3	Vicenin II	Flavonoid glycoside	C_27_H_30_O_15_	20.742	0.27	82.8	0.636
A5	Isomeranzin	Coumarin	C_15_H_16_O_4_	32.359	0.60	89.9	0.601
A4	Calycosin	Isoflavone	C_16_H_12_O_5_	27.015	−0.04	91.2	0.578
B5	Morin	Flavonol	C_15_H_10_O_7_	22.870	0.90	87.8	0.561
B3	Caffeic acid	Phenolic acid	C_9_H_8_O_4_	20.969	−0.86	88.5	0.541

*Note:* Peak labels refer to the corresponding peaks in total ion chromatograms in positive (A) and negative (B) ion modes (Figure 1A,B). Relative content was calculated based on peak area, indicated by the percentage of total area of the identified constituents.

Abbreviation: *R*_t_, retention time.

### 3.2 JWXZ Attenuates HFD‐Induced Weight Gain and Adipose Tissue Remodeling

**FIGURE 2:**
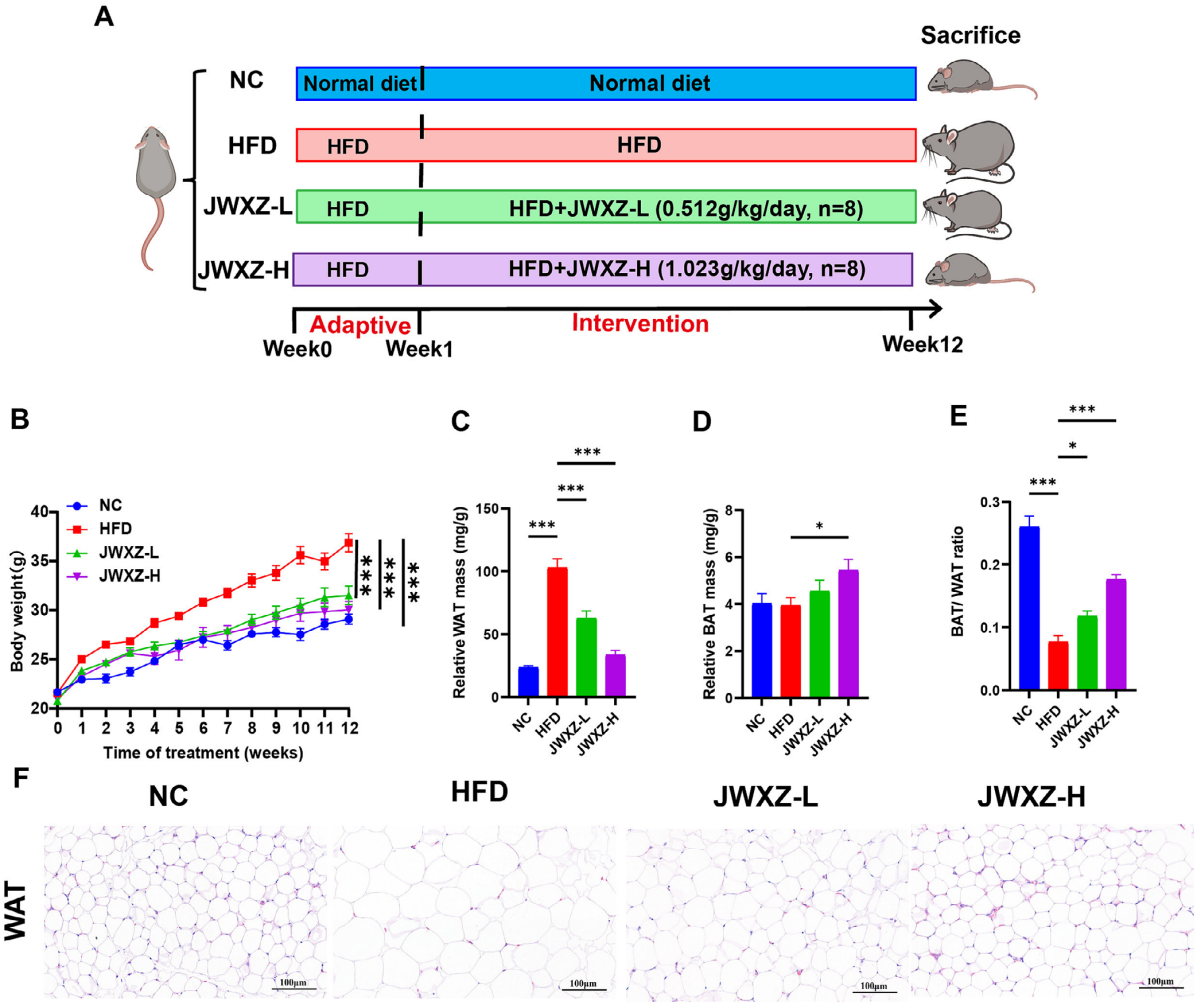
JWXZ attenuates HFD‐induced weight gain and adipose tissue remodeling. (A) Schematic of the experimental design. (B) Body weight changes during the 12‐week intervention. (C) Relative WAT mass. (D) Relative BAT mass. (E) BAT/WAT ratio. (F) Representative H&E images of WAT (scale bars = 100 μm). Data are presented as mean ± SD (*n* = 8 per group). *p < 0.05, **p < 0.01, ***p < 0.001 (compared with the HFD group). Data that follow a normal distribution with homogeneous variance are analyzed using one‐way ANOVA; otherwise, nonparametric tests are employed.

The HFD‐fed mice were used to evaluate how JWXZ affected body weight progression and adipose tissue remodeling. The experiment flowchart is shown in Figure 2A. HFD feeding induced an obesity‐like phenotype, as reflected by progressive body weight gain within a 12‐week experimental process. The HFD mice showed markedly increased body weight compared with NC mice, whereas JWXZ treatment attenuated HFD‐induced weight gain, and the JWXZ‐H group exhibited a superior effect (Figure 2B).

In line with the alterations in body weight, HFD feeding had a significant effect on the relative mass of WAT compared with the NC group. JWXZ treatment decreased the white adipose accumulation caused by HFD, with an even more obvious effect in the high‐dose group (Figure 2C). Although relative BAT mass was not markedly reduced by HFD feeding, JWXZ treatment increased relative BAT mass, particularly in the JWXZ‐H group (Figure 2D). Simultaneously, the BAT/WAT ratio was substantially decreased in HFD‐fed mice and was recovered after JWXZ treatment, indicating a better balance in the distribution of adipose tissue and energy metabolism (Figure 2E).

The results were validated by histological examination of WAT. The NC group exhibited comparatively small, evenly distributed adipocytes, and HFD feeding resulted in significant adipocyte hypertrophy. JWXZ treatment alleviated HFD‐induced adipocyte enlargement, with the JWXZ‐H group showing a more evident improvement in adipocyte morphology (Figure 2F). These findings suggest that JWXZ alleviates the HFD‐mediated obesity‐related adipose tissue remodeling, which is important for its positive effects on systemic metabolic dysfunction in MASLD.

### 3.3 JWXZ Improves Glucose‐Lipid Homeostasis and Attenuates Hepatic Steatosis in HFD‐Fed Mice

**FIGURE 3:**
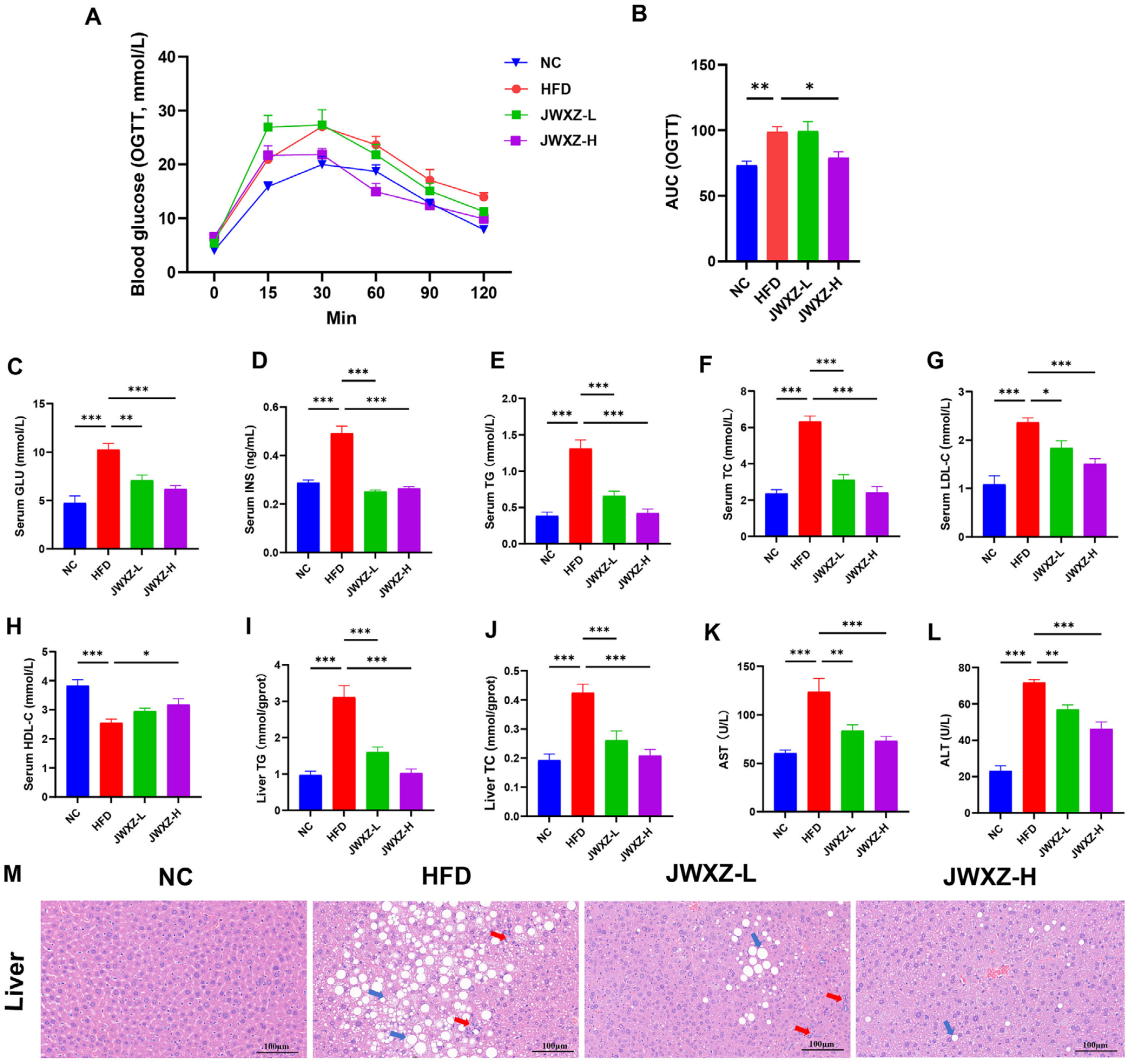
JWXZ improves glucose‐lipid homeostasis and attenuates hepatic steatosis in HFD‐fed mice. (A) OGTT curves. (B) AUC of OGTT. (C, D) Serum glucose and insulin. (E–H) Serum TG, TC, LDL‐C, and HDL‐C. (I, J) Hepatic TG and TC. (K, L) Serum AST and ALT. (M) Representative liver H&E images (scale bars = 100 μm). Data are presented as mean ± SD (*n* = 8 per group). *p < 0.05, **p < 0.01, ***p < 0.001 (compared with the HFD group). Data that follow a normal distribution with homogeneous variance are analyzed using one‐way ANOVA; otherwise, nonparametric tests are employed.

Subsequently, glucose homeostasis, lipid metabolic parameters, and hepatic injury markers were measured to assess whether the adiposity improvements were accompanied by more extensive metabolic advantages. The results of OGTT indicated that HFD dietary intake disrupted glucose metabolism, as indicated by high blood glucose levels following glucose loading and a much higher AUC than the NC group (Figure 3A,B). JWXZ therapy enhanced glucose tolerance, and a greater decrease in OGTT AUC was seen in the JWXZ‐H group (Figure 3A,B). Regularly, the fasting serum glucose and insulin levels substantially increased among mice fed on HFD, and the levels of both parameters were decreased by JWXZ, which means that the HFD‐induced hyperinsulinemia and hyperglycemia were improved (Figure 3C,D). These changes are in line with the common metabolic phenotype of HFD‐induced murine MASLD models, which are usually characterized by obesity, glucose intolerance, hyperinsulinemia, as well as hepatic steatosis (Miao et al. 2025).

HFD feeding also caused severe dyslipidemia. Relative to NC mice, HFD mice had significantly increased serum TG, TC, and LDL‐C contents, and decreased HDL‐C contents (Figure 3E–H). These serum lipid abnormalities were reversed by JWXZ treatment to different degrees, with the high‐dose group tending to exhibit a greater corrective effect (Figure 3E–H). Simultaneously, hepatic TC and TG contents dramatically increased due to HFD feeding and significantly decreased after JWXZ treatment, indicating that JWXZ mitigated hepatic lipid deposition (Figure 3I,J).

AST and ALT activities in serum were also measured to determine hepatic damage. HFD feeding greatly enhanced the activities of both transaminases, and JWXZ administration decreased AST and ALT contents, with the most significant effects being in the JWXZ‐H group (Figure 3K,L). These biochemical findings were consistent with histopathological analysis. For the NC group, its liver sections had normal liver structure, with ordered hepatocytes but with no lipid vacuolation. Conversely, liver tissues of HFD‐fed mice exhibited significant hepatic steatosis, which is extensive vacuolar degeneration and large lipid droplets. These pathological alterations were significantly alleviated by JWXZ treatment, which was shown by less lipid vacuolation and better hepatic histological morphology, especially in high‐dose mice (Figure 3M). Collectively, JWXZ enhances glucose and lipid metabolic homeostasis, decreases hepatic lipid buildup, and alleviates hepatic injury and steatosis caused by HFD.

### 3.4 Hepatic Transcriptomics Reveals That JWXZ Reprograms HFD‐Induced Cholesterogenic, ER‐Proteostatic, and Lipid‐Handling Transcriptional Programs

**FIGURE 4:**
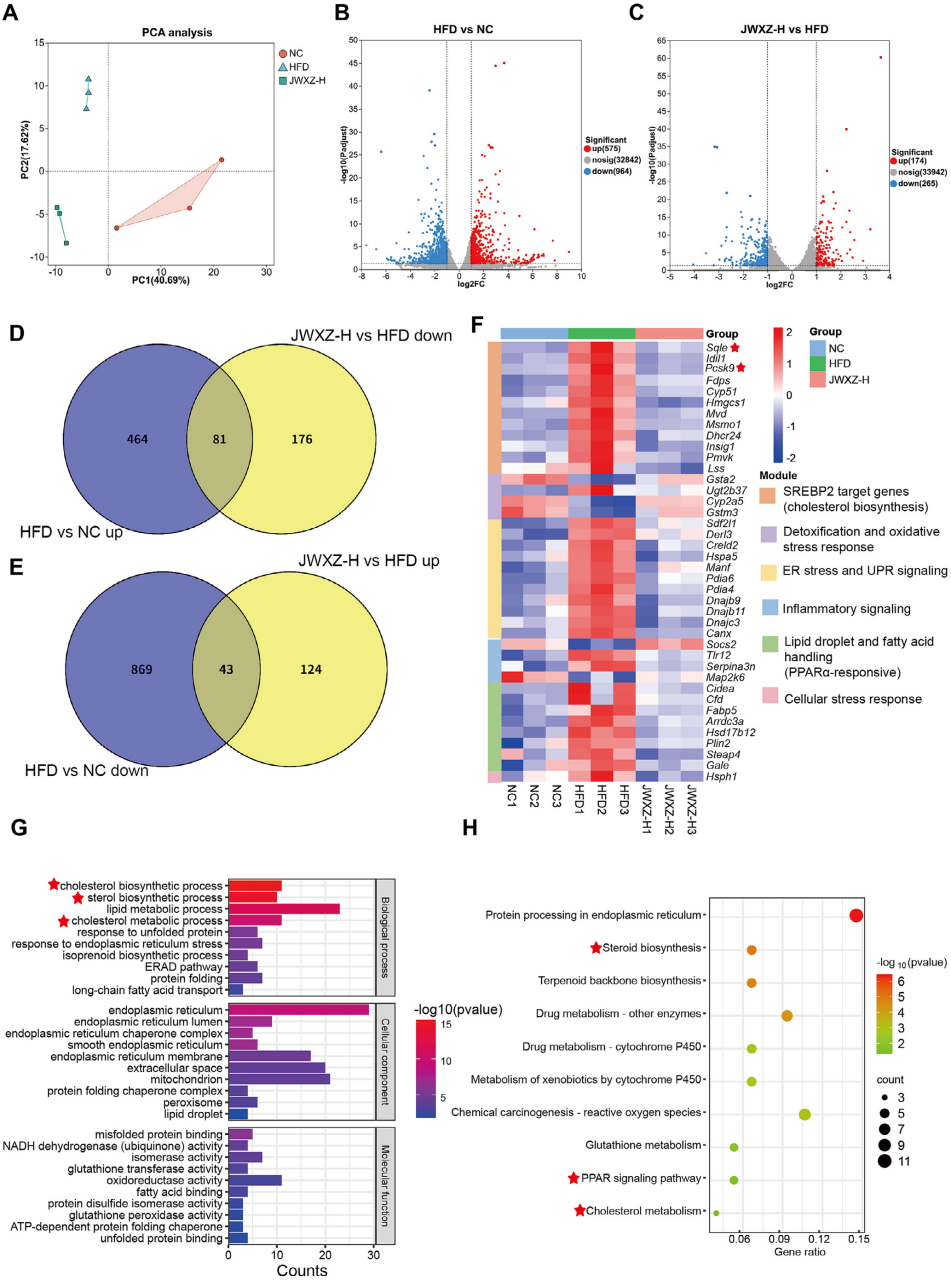
Hepatic transcriptomics reveals that JWXZ reprograms HFD‐induced cholesterogenic, ER‐proteostatic, and lipid‐handling transcriptional programs. (A) PCA of liver transcriptomes from NC, HFD, and JWXZ‐H groups. (B, C) Volcano plots of DEGs in HFD vs. NC and JWXZ‐H vs. HFD comparisons. (D, E) Venn diagrams showing reversed DEGs. (F) Heatmap of representative reversed DEGs grouped by functional modules. (G) GO enrichment of reversed DEGs. (H) KEGG enrichment of reversed DEGs. *n* = 3 per group.

To define the hepatic transcriptional changes associated with the hepatoprotective effects of JWXZ, RNA sequencing was performed on liver samples in NC, HFD, and JWXZ‐H groups (*n* = 3 per group). Principal component analysis showed that these three groups were clearly separated, and PC1 accounted for 40.69% of the total variance, indicating that HFD feeding markedly altered the hepatic transcriptome and that JWXZ‐H treatment partially reshaped the HFD‐associated expression profile (Figure 4A).

Using an adjusted *p* < 0.05 and |log_2_FC| ≥ 1 as the threshold, we identified 1539 DEGs in HFD versus NC groups, including 575 upregulated and 964 downregulated genes (Figure 4B). In the JWXZ‐H versus HFD comparison, 439 DEGs were detected, comprising 174 upregulated and 265 downregulated genes (Figure 4C). To identify HFD‐induced transcriptional abnormalities reversed by JWXZ‐H, oppositely regulated DEGs from the two comparisons were cross‐matched. This analysis identified 124 reversed DEGs, including 81 HFD‐upregulated/JWXZ‐H‐downregulated genes and 43 HFD‐downregulated/JWXZ‐H‐upregulated genes (Figure 4D,E).

A heatmap of representative reversed DEGs showed that HFD‐fed mice exhibited a distinct transcriptional pattern compared with NC mice, whereas JWXZ‐H administration shifted expression toward the NC pattern (Figure 4F). These reversed genes were functionally associated with SREBP2‐related cholesterol biosynthesis, detoxification and oxidative stress responses, ER stress and unfolded protein response signaling, inflammatory signaling, lipid droplet and fatty acid handling, and cellular stress responses. Notably, several cholesterogenic genes, including *Sqle*, *Idi1*, *Pcsk9*, *Fdps*, *Cyp51*, *Hmgcs1*, *Mvd*, *Msmo1*, *Dhcr24*, *Insig1*, *Pmvk*, and *Lss*, were induced by HFD feeding and suppressed by JWXZ‐H treatment, suggesting inhibition of the HFD‐activated hepatic cholesterol biosynthetic program.

According to GO analysis, the reversed DEGs were mostly related to cholesterol biosynthetic process, sterol biosynthetic process, lipid metabolic process, response to unfolded protein, response to ER stress, and protein folding (Figure 4G). Consistently, KEGG pathway analysis highlighted protein processing in the ER, steroid biosynthesis, terpenoid backbone biosynthesis, cholesterol metabolism, glutathione metabolism, chemical carcinogenesis‐reactive oxygen species, and the PPAR signaling pathway (Figure 4H). Collectively, these findings indicate that JWXZ‐H counteracts HFD‐induced hepatic transcriptional dysregulation by modulating SREBP2‐associated cholesterol biosynthesis, ER proteostatic stress, and PPARα‐related lipid handling. These results provide the basis for subsequent network pharmacology, molecular docking, and protein‐level validation.

### 3.5 Integration of Network Pharmacology and Transcriptomics Highlights Hepatic Lipid Metabolic Pathways

**FIGURE 5:**
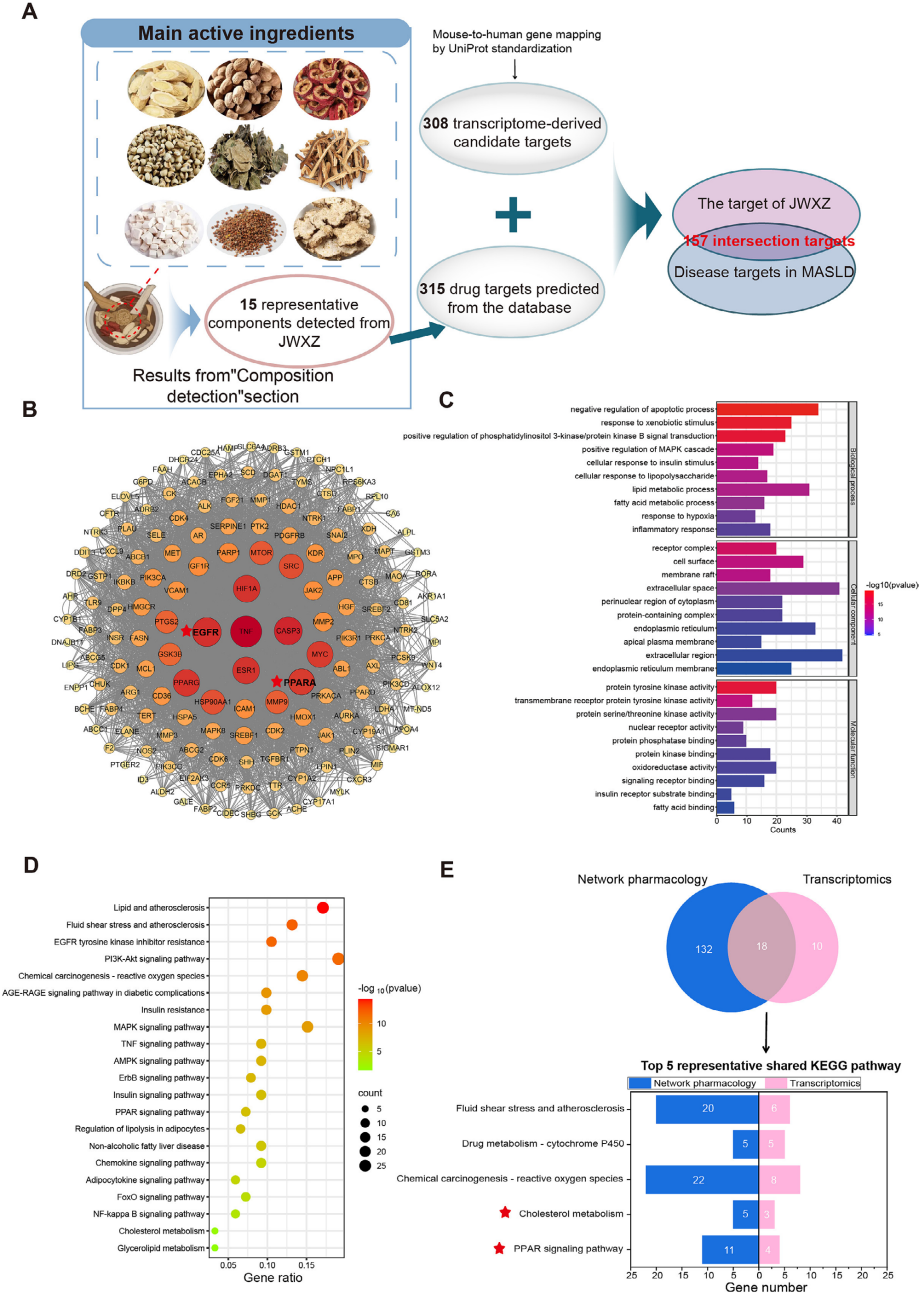
Integrated network pharmacology identifies potential JWXZ targets and pathways in MASLD. (A) Schematic workflow integrating JWXZ‐derived compound targets, transcriptome‐derived targets, and MASLD‐related disease targets. (B) PPI network. (C) Representative GO enrichment. (D) Representative KEGG enrichment. (E) Overlapping KEGG pathways between network pharmacology and transcriptomics.

Building on the representative compounds identified by UHPLC‐HRMS/MS, target prediction was performed to explore the potential pharmacological network of JWXZ against MASLD. After mouse‐to‐human gene mapping and UniProt standardization, 308 transcriptome‐derived candidate targets were obtained. In parallel, 315 compound‐related targets were predicted from public databases. The intersection of JWXZ‐predicted compound targets, transcriptome‐derived candidate targets, and curated MASLD‐related disease targets yielded 157 shared targets, which were subjected to subsequent network analysis (Figure 5A).

The PPI network constructed from these intersecting targets revealed several highly connected nodes, with EGFR and PPARα emerging as key hubs of particular mechanistic relevance to MASLD, alongside other prominent nodes including TNF, CASP3, SRC, MYC, MTOR, HIF1A, and PTGS2 (Figure 5B). Based on GO analysis, such targets were mostly related to lipid and fatty acid metabolic processes, response to xenobiotic stimulus, cellular response to insulin and lipopolysaccharide, inflammatory response, and kinase‐related molecular functions (Figure 5C).

KEGG enrichment analysis identified multiple MASLD‐related pathways, including those governing hepatic lipid metabolism (PPAR signaling pathway, cholesterol metabolism, glycerolipid metabolism, lipid and atherosclerosis, and non‐alcoholic fatty liver disease), insulin and growth factor pathway (PI3K‐Akt signaling, insulin signaling, insulin resistance, AMPK signaling, and ErbB signaling), and inflammatory or stress responses (MAPK signaling, TNF signaling, AGE‐RAGE signaling, and chemical carcinogenesis‐reactive oxygen species) (Figure 5D). To further prioritize biologically relevant pathways, KEGG terms from network pharmacology were compared with those from transcriptomic enrichment analysis. Eighteen pathways were shared between the two datasets, among which cholesterol metabolism and the PPAR signaling pathway were directly linked to transcriptomic signatures of SREBP2‐associated cholesterol biosynthesis and PPARα‐related lipid handling (Figure 5E). These findings supported the selection of SREBP2/HMGCR/SQLE‐mediated cholesterol biosynthesis, PPARα‐mediated FAO, and EGFR‐associated inflammatory signaling as three key mechanistic axes potentially related to the protection of JWXZ on HFD‐related MASLD. These pathways were subsequently validated at the protein level (Sections 3.6, 3.8), and their chemical basis was further explored through molecular docking (Section 3.9).

### 3.6 JWXZ Suppresses Hepatic SREBP2‐Mediated Cholesterol Biosynthesis

**FIGURE 6:**
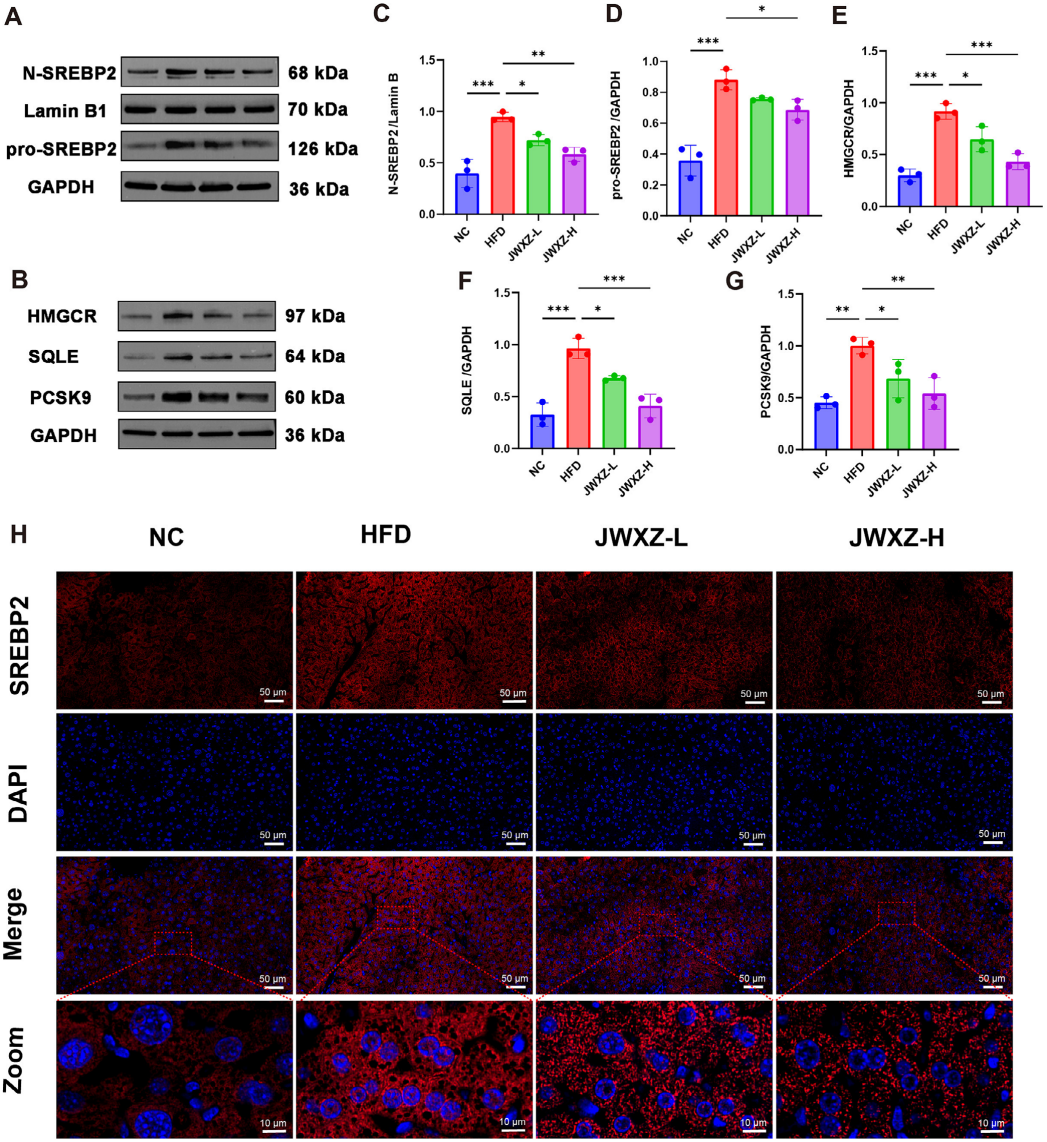
JWXZ suppresses hepatic SREBP2‐mediated cholesterol biosynthesis. (A, B) Representative Western blotting bands of N‐SREBP2, pro‐SREBP2, HMGCR, SQLE, and PCSK9. (C–G) Quantification of the corresponding protein levels. (H) Representative immunofluorescence images of hepatic SREBP2 (red) with DAPI‐counterstained nuclei (scale bars = 50 μm; 10 μm in enlarged images). Data are presented as mean ± SD (*n* = 8 per group). *p < 0.05, **p < 0.01, ***p < 0.001 (compared with the HFD group). Data that follow a normal distribution with homogeneous variance are analyzed using one‐way ANOVA; otherwise, nonparametric tests are employed.

To examine the SREBP2 axis identified through convergent transcriptomic and network pharmacology analyses, hepatic SREBP2 activation and its downstream effectors were assessed by Western blotting and immunofluorescence (Figure 6).

SREBP2 activation depends on sterol‐regulated proteolytic processing. Under conditions of cholesterol sufficiency, SREBP2 is retained in the ER as a 126 kDa precursor (pro‐SREBP2), whereas sterol depletion or metabolic stress triggers sequential cleavage to release a 68 kDa N‐terminal fragment (nuclear SREBP2, N‐SREBP2), and its nuclear translocation can drive cholesterogenic gene transcription. HFD feeding significantly enhanced the pro‐SREBP2 precursor and nuclear fragment in liver tissues, which showed persistent proteolytic activation of SREBP2 in chronic lipid overload (Figure 6A,C,D). JWXZ reversed this activation in a dose‐dependent fashion, decreasing both forms to NC levels, and the strongest effect was seen in the JWXZ‐H group.

In line with decreased nuclear SREBP2, HFD feeding significantly increased HMGCR, SQLE, and PCSK9, three canonical SREBP2 downstream targets in cholesterol biosynthesis and LDL receptor regulation. These changes were gradually reduced by JWXZ treatment at different doses (Figure 6B,E–G). These protein‐level results replicated the transcriptomic downregulation of cholesterol biosynthesis‐related genes in Section 3.4, suggesting a concerted action of JWXZ on the SREBP2 transcriptional program rather than selective inhibition of individual downstream enzymes.

These observations were further supported by immunofluorescence staining, which revealed weak SREBP2 signal in NC livers, strong and diffusely distributed immunoreactivity in HFD livers, and a progressive decrease after JWXZ treatment, with the JWXZ‐H group resembling the NC pattern (Figure 6H).

Overall, JWXZ inhibits hepatic SREBP2 signaling by reducing the pool of precursors and the formation of the transcriptionally active nuclear form, which leads to the downregulation of downstream cholesterogenic effectors, such as HMGCR, SQLE, and PCSK9.

### 3.7 JWXZ Restores Hepatic PPARα‐Mediated FAO

**FIGURE 7:**
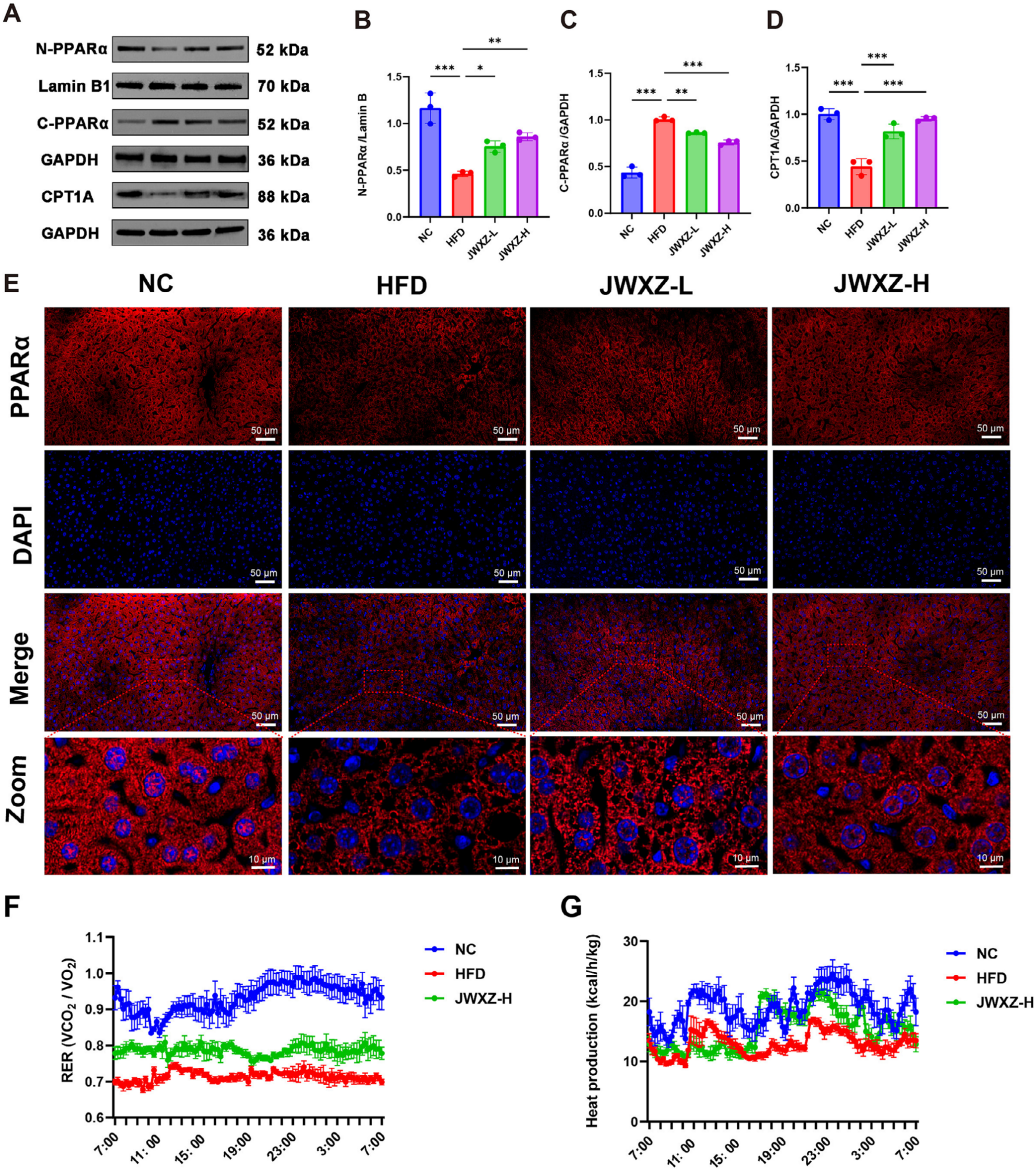
JWXZ restores hepatic PPARα signaling and improves energy metabolism in HFD‐fed mice. (A) Representative Western blotting bands of nuclear PPARα, cytoplasmic PPARα, and CPT1A. (B–D) Quantification of the corresponding protein levels. (E) Representative immunofluorescence images of hepatic PPARα (red) with DAPI‐counterstained nuclei (scale bars = 50 μm; 10 μm in enlarged images). (F, G) RER and heat production measured by indirect calorimetry. Data are presented as mean ± SD (*n* = 8 per group). *p < 0.05, **p < 0.01, ***p < 0.001 (compared with the HFD group). Data that follow a normal distribution with homogeneous variance are analyzed using one‐way ANOVA; otherwise, nonparametric tests are employed.

The activation state and downstream response of hepatic PPARα signaling were measured by Western blotting and immunofluorescence to validate the PPARα axis detected through network pharmacology combined with molecular docking analyses (Figure 7).

Nuclear translocation is a ligand‐regulated activity of PPAR. PPARα is mainly found in the cytoplasmic compartment (C‐PPARα) in the basal state, but the binding of ligands facilitates its nuclear translocation (N‐PPARα) to control FAO gene expression. HFD feeding significantly decreased nuclear PPARα and simultaneously elevated the cytoplasmic fraction, which showed that nuclear translocation was impaired with chronic lipid overload (Figure 7A–C). This redistribution was reversed in a dose‐dependent fashion by JWXZ treatment, which enhanced N‐PPARα and reduced C‐PPARα, with the JWXZ‐H group being most similar to the NC profile.

In line with the recovered nuclear PPARα levels, CPT1A, a rate‐limiting enzyme in mitochondrial long‐chain fatty acid β‐oxidation, a canonical PPARα target, was significantly inhibited by HFD and dose‐dependently recovered by JWXZ, and the JWXZ‐H group restored CPT1A expression to near‐NC levels (Figure 7A,D). Immunofluorescence staining also confirmed that the hepatic PPARα signal was suppressed in the HFD liver and gradually recovered after JWXZ treatment. The enrichment of nuclear PPARα in the NC and JWXZ‐H groups was prominent in high‐magnification images, which were in line with the results of the Western blotting (Figure 7E).

Indirect calorimetry at the whole‐body level showed that HFD‐fed mice had a consistently low RER near 0.70, indicating that the HFD caused the use of lipid substrates as the primary energy source and decreased metabolic flexibility. JWXZ‐H treatment had a significant effect on RER to intermediate values, which means that the substrate switching capacity was partially restored, and no additional increase in fixed lipid oxidation (Figure 7F). The JWXZ‐H group was also found to produce more heat during the dark phase, which indicated a better overall energy expenditure (Figure 7G).

Overall, these findings indicate that JWXZ is associated with restored hepatic PPARα signaling, reflected by increased nuclear translocation and recovery of downstream FAO, accompanied by improved systemic metabolic flexibility.

### 3.8 JWXZ Attenuates Hepatic EGFR‐Mediated Inflammatory Signaling

**FIGURE 8:**
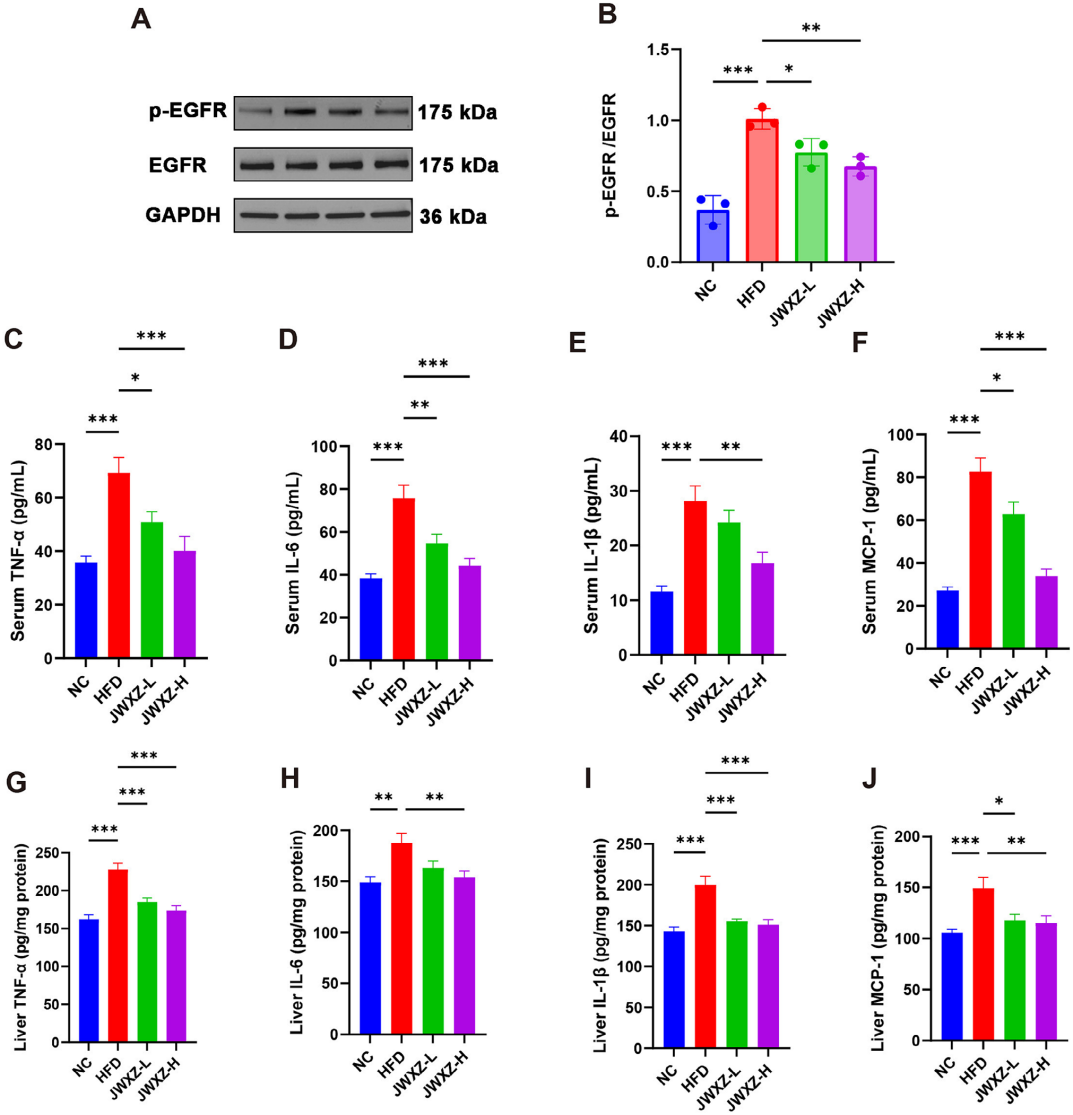
JWXZ attenuates hepatic EGFR‐mediated inflammatory signaling. (A) Representative Western blotting bands of p‐EGFR and total EGFR in liver. (B) Quantification of the p‐EGFR/EGFR ratio. (C–F) Serum levels of TNF‐α, IL‐6, IL‐1β, and MCP‐1. (G–J) Hepatic levels of TNF‐α, IL‐6, IL‐1β, and MCP‐1. Data are presented as mean ± SD (*n* = 8 per group). *p < 0.05, **p < 0.01, ***p < 0.001 (compared with the HFD group). Data that follow a normal distribution with homogeneous variance are analyzed using one‐way ANOVA; otherwise, nonparametric tests are employed.

To explore the EGFR‐linked inflammatory pathway discovered by the integrated network pharmacology analysis, hepatic EGFR activation and downstream inflammatory biomarkers were analyzed in liver tissues and serum (Figure 8).

EGFR is a receptor tyrosine kinase, the activation of which in metabolic stress converts into several downstream cascades (e.g., MAPK and PI3K‐AKT signaling) to stimulate hepatic inflammatory responses; therefore, phosphorylated EGFR (p‐EGFR) is a sensitive marker of receptor activation. Western blotting showed that HFD feeding significantly enhanced the hepatic p‐EGFR/EGFR ratio, which indicates that EGFR signaling is persistently activated in chronic lipid overload (Figure 8A,B). The p‐EGFR/EGFR ratio decreased dose‐dependently, and the JWXZ‐H group had a significant decrease as compared to the HFD group.

TNF‐α, IL‐6, IL‐1β, and MCP‐1 levels within liver tissue and serum were assessed as inflammatory responses. HFD feeding significantly enhanced all four cytokines in hepatic tissues, but JWXZ treatment significantly reduced these changes, with the most significant decrease in the JWXZ‐H group (Figure 8G–J). Similar results were found in serum, with HFD‐induced increases in circulating TNF‐α, IL‐6, IL‐1β, and MCP‐1 dose‐dependently inhibited after JWXZ administration (Figure 8C–F), suggesting that JWXZ's anti‐inflammatory efficacy was not confined to the hepatic compartment.

Given the marked suppression of the SREBP2‐mediated cholesterogenic program and the restoration of PPARα‐mediated FAO, the attenuation of EGFR‐associated inflammatory signaling appears to be primarily linked to improved hepatic lipid homeostasis rather than direct receptor inhibition. This interpretation is further supported by the molecular docking results presented in Section 3.9.

### 3.9 Molecular Docking Suggests a Potential Compound‐Target Basis for the Validated Metabolic Axes

**FIGURE 9:**
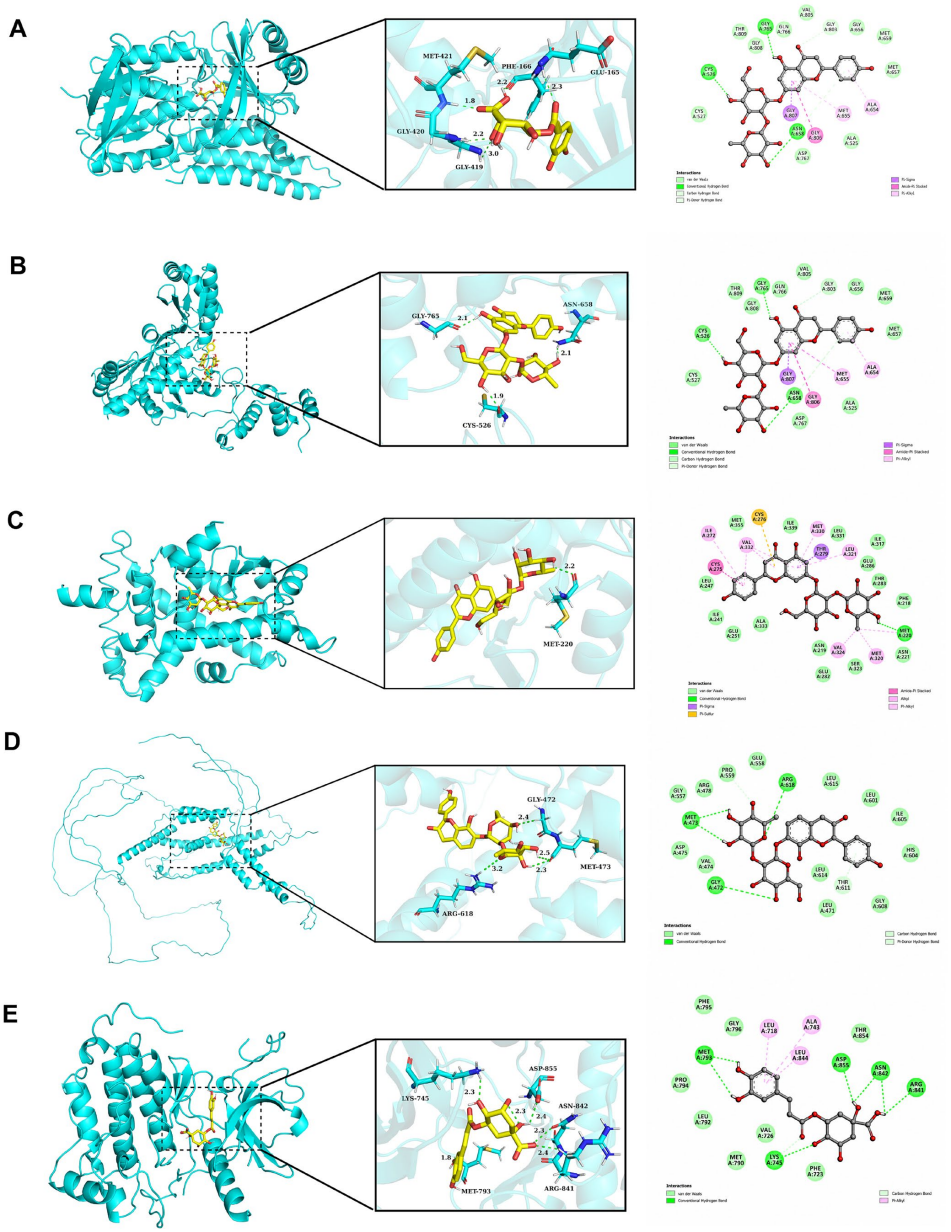
Representative molecular docking models of JWXZ‐derived compounds with selected targets. (A) SQLE‐chlorogenic acid. (B) HMGCR‐naringin. (C) PPARα‐rhoifolin. (D) SREBP2‐rhoifolin. (E) EGFR‐chlorogenic acid. For each panel, the overall docked pose (left), a magnified view of the binding pocket with key interacting residues (middle), and the corresponding 2D interaction diagram (right) are shown.

After experimental validation of the SREBP2‐mediated cholesterogenic axis, the PPARα‐mediated FAO axis, and EGFR‐associated inflammatory signaling, molecular docking studies were conducted for exploring the possible compound‐target interactions underlying these effects (Table 2, Figure 9).

**TABLE 2:** Representative molecular docking results of JWXZ‐derived bioactive constituents with key target proteins of the validated metabolic axes.

Target	Structure ID	Compound	Binding affinity (kcal/mol)	Mechanistic relevance
SQLE	PDB: 6C6N	Chlorogenic acid	−9.4	Cholesterol biosynthesis
SQLE	PDB: 6C6N	Naringenin chalcone	−9.0	Cholesterol biosynthesis
SQLE	PDB: 6C6N	Calycosin	−8.6	Cholesterol biosynthesis
HMGCR	PDB: 2R4F	Naringin	−8.4	Cholesterol biosynthesis
HMGCR	PDB: 2R4F	Rhoifolin	−8.0	Cholesterol biosynthesis
HMGCR	PDB: 2R4F	Calycosin	−7.5	Cholesterol biosynthesis
PPARα	PDB: 6KAX	Rhoifolin	−8.7	Fatty acid oxidation
PPARα	PDB: 6KAX	Calycosin‐7‐O‐β‐D‐glucoside	−8.6	Fatty acid oxidation
PPARα	PDB: 6KAX	Calycosin	−8.1	Fatty acid oxidation
PPARα	PDB: 6KAX	Isoquercitrin	−8.0	Fatty acid oxidation
SREBP2	UniProt: Q12772	Rhoifolin	−7.4	SREBP2‐associated cholesterogenic regulation
EGFR	PDB: 6LUD	Chlorogenic acid	−7.2	EGFR‐associated inflammatory signaling

*Note:* Binding affinities were calculated using AutoDock Vina, expressed as the lowest‐energy docking conformations in kcal/mol; more negative values suggest more potent predicted binding. Target structures were obtained based on the Protein Data Bank (PDB) or, in the absence of an experimental structure, from the AlphaFold Protein Structure Database ( *Homo sapiens* ). Compound identification and abundance information are provided in Table 1. Complete docking results for all tested compound‐target pairs are listed in Table S3.

For the cholesterol biosynthesis axis, SQLE showed favorable predicted binding with several JWXZ‐derived constituents, including chlorogenic acid, naringenin chalcone, and calycosin, with docking scores of −9.4, −9.0, and −8.6 kcal/mol, respectively. HMGCR also exhibited favorable predicted interactions with naringin, the most abundant representative constituent identified in JWXZ, as well as with rhoifolin, with docking scores of −8.4 and −8.0 kcal/mol, separately. Additionally, rhoifolin demonstrated the strongest predicted binding affinity for SREBP2 among the tested compounds, and its docking score was −7.4 kcal/mol. These results suggest that phenolic acids and flavonoid‐related constituents may contribute to the modulation of the SREBP2‐associated cholesterogenic axis.

For the FAO axis, PPARα showed favorable predicted interactions with multiple flavonoid constituents, particularly rhoifolin, calycosin‐7‐O‐β‐D‐glucoside, and calycosin, with docking scores of −8.7, −8.6, and −8.1 kcal/mol, separately. Representative docking models indicated that these ligands were accommodated within the predicted binding pockets through hydrogen bonding, hydrophobic interactions, and π‐related contacts (Figure 9A–D). These results offer computational evidence of a possible role of flavonoid compounds in PPARα‐mediated lipid metabolic control.

The affinity of the JWXZ‐derived constituents to EGFR was less pronounced compared to the targets related to lipid metabolism. Chlorogenic acid showed the strongest predicted interaction with EGFR, and its docking score was −7.2 kcal/mol (Figure 9E). This result suggests that the reduction in hepatic p‐EGFR and inflammatory cytokines observed following JWXZ treatment may not be explained solely by direct compound‐EGFR binding, but may also reflect indirect attenuation secondary to improved hepatic lipid homeostasis and reduced metabolic stress.

Overall, these docking outcomes must be explained as supportive computational evidence but not definitive evidence supporting direct target involvement. Nevertheless, they provide a plausible compound‐target framework linking key chemical constituents of JWXZ, particularly chlorogenic acid, naringin, rhoifolin, naringenin chalcone, and calycosin‐related compounds, with the validated metabolic and inflammatory pathways in HFD‐induced MASLD. The interactions predicted by the aforementioned molecular docking studies require further validation through direct binding assays, enzyme activity assays, or cellular experiments. The complete molecular docking results for all tested compound‐target pairs are listed in Table S3.

## 4 Discussion

This study provides an integrated chemical and mechanistic evaluation of JWXZ in HFD‐induced MASLD. JWXZ improved body weight gain, adipose tissue remodeling, glucose intolerance, dyslipidemia, hepatic lipid accumulation, and liver injury. Mechanistically, chemical profiling, transcriptomics, network pharmacology, molecular docking, and protein‐level validation converged on three closely interconnected regulatory axes: suppression of SREBP2‐associated cholesterol biosynthesis, restoration of PPARα‐mediated FAO, and attenuation of EGFR‐associated inflammatory signaling. These findings suggest that JWXZ ameliorates MASLD not through a single isolated target, but via coordinated modulation of hepatic lipid metabolism and inflammatory stress.

Via UHPLC‐HRMS/MS analysis, 178 chemical constituents were identified in JWXZ. Among these constituents, 15 representative bioactive compounds were selected for target prediction, and naringin, sinapine, naringenin chalcone, chlorogenic acid, and corchorifatty acid F were detected at relatively high abundances. Several of these compounds have been reported to possess lipid‐lowering and hepatoprotective properties. An example is that naringin has been demonstrated to inhibit hepatic cholesterol synthesis and alleviate lipid metabolic disorders in experimental fatty liver disease models (Pengnet et al. 2022; Sun, Xue, and Zhang 2025). Likewise, chlorogenic acid has been widely investigated in terms of its effects on suppressing oxidation, inflammation, and metabolic regulation in MASLD (Nguyen et al. 2024; Ziółkiewicz et al. 2024). Our molecular docking results also revealed positive binding affinities of naringin to HMGCR, chlorogenic acid to SQLE and EGFR, and rhoifolin to PPARα and SREBP2. Though these results offer initial structural evidence of possible compound‐target interactions, they cannot be considered as conclusive evidence of direct target interaction. Nevertheless, the docking results conform to the multi‐component, multi‐target pharmacological features of JWXZ and support the proposed mechanisms underlying its therapeutic effects.

The current results point to the inhibition of the cholesterogenic axis of SREBP2 as one of the main mechanisms of the protection of JWXZ on MASLD in HFD. As revealed by transcriptomic analysis, a HFD dramatically increased coordinated levels of cholesterol and sterol biosynthesis genes, like *Sqle*, *Pcsk9*, *Hmgcs1*, *Cyp51*, *Mvd*, *Msmo1*, *Pmvk*, and *Lss*. JWXZ‐H treatment significantly reversed this transcriptional program. JWXZ decreased the expression of the precursor form of SREBP2 (pro‐SREBP2) and its proteolytically cleaved nuclear form (N‐SREBP2) at the protein level; in addition, HMGCR, SQLE, and PCSK9 levels were reduced subsequently. The SCAP/SREBP2 axis is a key transcriptional controller of hepatic cholesterol synthesis, and its pathological activation is becoming a hallmark of MASLD pathogenesis (Barbhuiya et al. 2025; Chandrasekaran and Weiskirchen 2024). Notably, the nuclear abundance of N‐SREBP2 is dynamically controlled by ubiquitin‐proteasome‐mediated degradation, and its stabilization promotes transcriptional activation of the mevalonate‐cholesterol biosynthetic pathway (Ozkan‐Nikitaras et al. 2024). The simultaneous reduction in pro‐SREBP2, N‐SREBP2, and their downstream cholesterogenic targets in JWXZ‐treated livers suggests that JWXZ suppresses hepatic cholesterol biosynthesis through multiple regulatory mechanisms rather than by targeting a single enzyme. Among these downstream effectors, SQLE, as the rate‐limiting enzyme during later stages of sterol biosynthesis, is an essential driving factor for nonalcoholic steatohepatitis initiation and progression. Indeed, hepatic SQLE overexpression is sufficient to induce steatohepatitis‐like phenotypes in mice (Liu et al. 2021). PCSK9, another well‐established SREBP2 target, contributes to MASLD progression by promoting low‐density lipoprotein receptor degradation and has been positively related to hepatic steatosis severity among obese individuals (Bao et al. 2024). Therefore, the coordinated downregulation of SREBP2, HMGCR, SQLE, and PCSK9 by JWXZ indicates a broad suppression of cholesterogenic signaling rather than a single‐target effect. This inhibition at the program level offers a reasonable molecular explanation of the decreases in hepatic and circulating cholesterol in the current model.

The PPARα‐associated results offer a second mechanistic stratum of the hepatic activities of JWXZ. HFD feeding resulted in a redistribution of hepatic PPARα to the cytoplasmic compartment, with a decrease in the nuclear pool and down‐regulation of CPT1A. These changes were associated with a decreased RER and reduced dark‐phase heat production. This trend was reversed by JWXZ treatment, which restored nuclear PPARα abundance, enhanced CPT1A expression, and partially restored systemic metabolic flexibility. As the ligand‐activated nuclear report, PPARα controls hepatic uptake of fatty acids, mitochondrial β‐oxidation, and ketogenesis by transcriptionally activating target genes, such as *Cpt1a*, *Acox1*, and *Ehhadh*. Its gradual functional deterioration has been more and more identified as a characteristic of MASLD progression (Carli et al. 2024). Importantly, PPARα transcriptional activity is critically dependent on its nuclear localization, which is dynamically regulated by RAN/CRM1‐dependent nucleocytoplasmic shuttling. This balance is impaired in NAFLD livers, resulting in cytoplasmic retention of PPARα and decreased transcriptional activity despite an increase in the availability of fatty acid‐derived ligands (Zhong et al. 2023). Recent research has also demonstrated that aberrant cytoplasmic sequestration of PPARα by FABP5 directly inhibits its binding to peroxisome proliferator response elements, thus inhibiting downstream FAO in MASLD (Yu et al. 2025). The recovery of nuclear PPARα and CPT1A with JWXZ, therefore, is probably due to reactivation of the nuclear translocation‐transcription axis, and not due to alterations in total PPARα abundance, which is in line with the Western blotting results showing subcellular redistribution, but not loss of expression. This PPARα‐mediated FAO recovery can be combined with inhibition of SREBP2‐regulated cholesterol biosynthesis, and thus, lipid synthesis and lipid clearance can be simultaneously decreased and increased, respectively. This type of coordinated regulation offers a plausible mechanistic explanation of the dramatically reduced hepatic TC and TG contents, and the partially recovered whole‐body metabolic flexibility in JWXZ‐treated mice.

The EGFR‐associated results give another connection between lipid metabolic stress and inflammatory activation in MASLD induced by HFD. Network pharmacology revealed EGFR as a hub in the inflammation‐related target module of JWXZ, and protein‐level analysis indicated that JWXZ decreased hepatic p‐EGFR/EGFR levels and reduced circulating and hepatic TNF‐α, IL‐6, IL‐1β, and MCP‐1 contents. The reduction of multiple inflammatory cytokines may also be interpreted in the context of the emerging cytokine‐epigenetic axis, in which cytokine signaling and epigenetic regulation reciprocally shape disease‐associated transcriptional programs (Luo et al. 2026). These findings conform to prior evidence that EGFR signaling is crucial for hepatic lipid dysregulation and metabolic inflammation caused by HFD. It has been reported that pharmacological inhibition of EGFR can reduce hepatic steatosis, hypercholesterolemia, and metabolic dysfunction in obese mice, in part by inhibiting PI3K/AKT signaling and cholesterol biosynthesis via SREBP (Choung et al. 2019). Recent work has placed EGFR as a mechanistic interface between hepatic lipotoxic stress and inflammation, where amphiregulin‐mediated EGFR signaling enhances profibrotic transcriptional programs in hepatic stellate cells and enhances immune cell recruitment in MASLD/MASH (He et al. 2025). However, in the current docking analysis, the binding affinities of the JWXZ‐derived compounds to EGFR were only predicted to be moderate (−4.5 to −7.2 kcal/mol), which was significantly lower than those of HMGCR, SQLE, and PPARα. The strong inhibition of p‐EGFR and downstream cytokine synthesis by JWXZ is thus unlikely to be due to direct receptor antagonism but rather an indirect effect of upstream metabolic reprogramming. This explanation is consistent with the established lipotoxicity‐inflammation nexus in MASLD, where free cholesterol, ceramide, and saturated fatty acid accumulation within the liver leads to ER stress and the activation of tyrosine kinase signaling cascades, such as EGFR‐MAPK, to stimulate cytokine production and immune cell recruitment (Carli et al. 2024; Venkatesan et al. 2023). JWXZ probably lowers the upstream lipotoxic load that maintains chronic EGFR phosphorylation by inhibiting SREBP2‐mediated cholesterogenesis and reinstating PPARα‐mediated FAO. The reported inhibition of p‐EGFR and inflammatory cytokines, therefore, seems to be a concerted downstream effect of enhanced hepatic metabolic homeostasis and not a receptor‐specific inhibition.

**FIGURE 10:**
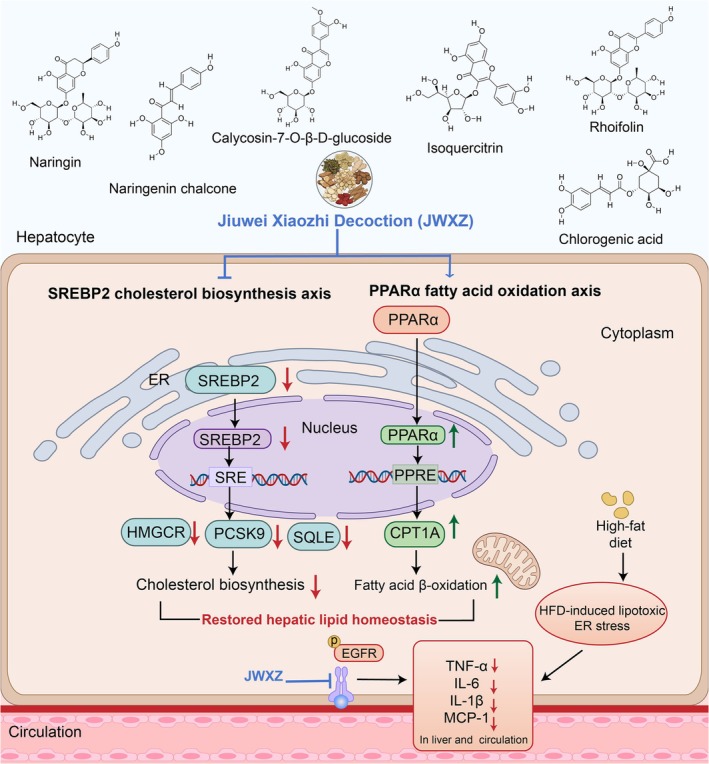
Mechanism by which JWXZ ameliorates HFD‐induced MASLD by rebalancing hepatic lipid metabolism. JWXZ suppresses SREBP2‐mediated cholesterol biosynthesis (downregulating HMGCR, SQLE, and PCSK9), restores PPARα‐mediated fatty acid oxidation (promoting PPARα nuclear translocation and CPT1A expression), and attenuates EGFR‐associated inflammatory signaling as a downstream consequence of restored hepatic lipid homeostasis. This figure also serves as the graphical abstract of the study.

The overall data of transcriptomic profiling, network pharmacology, molecular docking, and protein‐level validation indicates a concerted action in which HFD feeding increases hepatic cholesterogenesis, disrupts fatty acid oxidative metabolism, and enhances inflammatory signaling. JWXZ effectively counteracted these pathological alterations by suppressing SREBP2‐dependent cholesterol biosynthesis, restoring the PPARα‐mediated lipid catabolic pathway, and attenuating EGFR‐associated inflammatory responses (Figure 10). This mechanistic model aligns with the multifactorial pathogenesis of MASLD, where lipid metabolism, organelle stress, and chronic low‐grade inflammation disturbances are closely interrelated and mutually reinforcing. Taken together, these results indicate that JWXZ has its therapeutic effects by the concerted regulation of several metabolic and inflammatory pathways, which justify its use as a multi‐target intervention in MASLD.

Although these findings were made, there are a number of limitations that must be noted. Even though transcriptomic, immunofluorescence, and Western blotting analyses have consistently implicated the SREBP2 and PPARα signaling axes, the causal roles of these pathways in mediating the protective effects of JWXZ have not been clearly determined. Future research using hepatocyte‐specific knockdown or knockout models is justified to establish whether the metabolic benefits of JWXZ require modulation of these pathways. Furthermore, the molecular docking analyses provide only computational predictions of compound‐target interactions. Further experimental confirmation, such as direct binding assays, enzyme activity analyses, and cellular gain‐ and loss‐of‐function studies, is required to confirm the predicted molecular interactions and their biological significance. Given that post‐translational modifications such as SUMOylation can influence protein stability, localization, transcriptional activity, and disease‐associated signaling, future work should further assess whether SUMOylation‐related regulation contributes to the effects of JWXZ on SREBP2/PPARα‐linked metabolic remodeling (Wu et al. 2026). The MASLD model induced by HFD applied in the current research effectively replicates a number of the most important clinical and pathological characteristics of the disease, like obesity, hepatic steatosis, insulin resistance, and liver damage. Nonetheless, it does not fully reproduce the advanced fibrosis and pathological complexity observed in MASH. Therefore, further validation in fibrosis‐prone dietary models, genetically modified models, and human‐relevant experimental systems would enhance the translational significance of the present findings. In addition, the potential involvement of gut microbiota and bile acid signaling was not investigated. Given the emerging roles of these pathways in both MASLD pathogenesis and the pharmacological actions of traditional Chinese medicine, future studies exploring their contribution may help comprehensively understand those therapeutic mechanisms underlying JWXZ.

## 5 Conclusion

Collectively, the present work provides an integrated chemical and mechanistic characterization of JWXZ in an HFD‐induced MASLD model. By integrating UHPLC‐HRMS/MS profiling, in vivo pharmacological assessment, hepatic transcriptomic analysis, network pharmacology, molecular docking, and multi‐level protein validation, we demonstrated that JWXZ effectively ameliorates HFD‐induced MASLD through coordinated reprogramming of hepatic lipid metabolism. In particular, its therapeutic action entails three interrelated molecular processes: inhibition of SREBP2‐mediated cholesterol synthesis, reinstatement of PPARα‐dependent FAO, and consequent inhibition of EGFR‐linked inflammatory signaling. Moreover, naringin, chlorogenic acid, and rhoifolin were also found to be priority bioactive constituents that can play a significant role in these pharmacological effects. Taken together, these results provide a consistent chemical‐pharmacological rationale in favor of JWXZ as a multi‐component, multi‐target therapeutic approach to MASLD and a mechanistic explanation of why it should be further developed preclinically and studied in translational research.

## Author Contributions

**Youcheng Ma:** funding acquisition, formal analysis, investigation, software, methodology, writing – original draft, validation, visualization. **Zhen Huang:** formal analysis, funding acquisition, investigation, methodology, project administration, writing – original draft. **Fangli Li:** funding acquisition, methodology, project administration, resources, software, supervision, writing – review and editing. **Ji Wang:** data curation, methodology, writing – review and editing. **Xinrui Gao:** conceptualization, data curation, formal analysis, investigation, writing – original draft. **Lingru Li:** writing – review and editing, methodology, supervision, validation, visualization.

## Funding

The authors declare that financial support was received for the research, authorship, and/or publication of this article. This study was supported by the Medical and Health Three Famous Project (Sanming Project) of Shenzhen (SZZYSM202205008); the Natural Science Foundation of Shenzhen Municipality (JCYJ20230807150911023, JCYJ20240813165130040); and the Special Fund for Scientific and Technological Innovation of Shenzhen Longgang District (LGWJ2024‐29).

## Ethics Statement

The animal study was reviewed and approved by the Institutional Animal Care and Use Committee of Beijing University of Chinese Medicine (Approval No. BUCM20250729‐001). The study was conducted in accordance with local legislation and institutional requirements.

## Conflicts of Interest

The authors declare no conflicts of interest.

## Supporting information


**Figure S1:** Total ion chromatograms of the blank sample. (A) TIC of the blank sample acquired in positive ion mode. (B) TIC of the blank sample acquired in negative ion mode. The blank sample was prepared and analyzed under the same UHPLC‐HRMS/MS conditions as JWXZ to assess background signals and potential carry‐over interference.
**Table S1:** Detailed composition of the diets.
**Table S2:** Complete chemical constituents identified in JWXZ by UHPLC‐HRMS/MS.
**Table S3:** Complete molecular docking results.
**Table S4:** Antibodies used for Western blotting and immunofluorescence staining.

## Data Availability

The data that support the findings of this study are openly available in NCBI at https://www.ncbi.nlm.nih.gov/bioproject/PRJNA1475792, reference number PRJNA1475792.

## References

[cbdd70371-bib-0001] Ann, J. Y. , H. Eo , and Y. Lim . 2015. “Mulberry Leaves ( *Morus alba* L.) Ameliorate Obesity‐Induced Hepatic Lipogenesis, Fibrosis, and Oxidative Stress in High‐Fat Diet‐Fed Mice.” Genes and Nutrition 10, no. 6: 46. 10.1007/s12263-015-0495-x. 26463593 PMC4604156

[cbdd70371-bib-0002] Bao, X. , Y. Liang , H. Chang , et al. 2024. “Targeting Proprotein Convertase Subtilisin/Kexin Type 9 (PCSK9): From Bench to Bedside.” Signal Transduction and Targeted Therapy 9, no. 1: 13. 38185721 10.1038/s41392-023-01690-3 PMC10772138

[cbdd70371-bib-0003] Barbhuiya, P. A. , R. Yoshitomi , and M. P. Pathak . 2025. “Understanding the Link Between Sterol Regulatory Element Binding Protein (SREBPs) and Metabolic Dysfunction Associated Steatotic Liver Disease (MASLD).” Current Obesity Reports 14, no. 1: 36. 40227546 10.1007/s13679-025-00626-y

[cbdd70371-bib-0004] Carli, F. , G. Della Pepa , S. Sabatini , A. V. Puig , and A. Gastaldelli . 2024. “Lipid Metabolism in MASLD and MASH: From Mechanism to the Clinic.” JHEP Reports 6, no. 12: 101185. 39583092 10.1016/j.jhepr.2024.101185 PMC11582433

[cbdd70371-bib-0005] Chandrasekaran, P. , and R. Weiskirchen . 2024. “The Role of SCAP/SREBP as Central Regulators of Lipid Metabolism in Hepatic Steatosis.” International Journal of Molecular Sciences 25, no. 2: 1109. 38256181 10.3390/ijms25021109 PMC10815951

[cbdd70371-bib-0006] Cho, M. H. , H. Jin , J. Ha , S. Chu , and S. An . 2025. “An Integrated Systems Pharmacology Approach Combining Bioinformatics, Untargeted Metabolomics and Molecular Dynamics to Unveil the Anti‐Aging Mechanisms of Tephroseris Flammea.” Biomolecules 15, no. 12: 1740. 10.3390/biom15121740. 41463393 PMC12730562

[cbdd70371-bib-0007] Choung, S. , J. M. Kim , K. H. Joung , E. S. Lee , H. J. Kim , and B. J. Ku . 2019. “Epidermal Growth Factor Receptor Inhibition Attenuates Non‐Alcoholic Fatty Liver Disease in Diet‐Induced Obese Mice.” PLoS One 14, no. 2: e0210828. 30735525 10.1371/journal.pone.0210828 PMC6368280

[cbdd70371-bib-0008] European Association for the Study of the Liver , European Association for the Study of Diabetes , and European Association for the Study of Obesity . 2024. “EASL‐EASD‐EASO Clinical Practice Guidelines on the Management of Metabolic Dysfunction‐Associated Steatotic Liver Disease (MASLD): Executive Summary.” Diabetologia 67, no. 11: 2375–2392. 10.1007/s00125-024-06196-3. 38869512 PMC11519095

[cbdd70371-bib-0009] Fan, Y. , Q. Zhao , Y. Wei , et al. 2023. “Pingwei San Ameliorates Spleen Deficiency‐Induced Diarrhea Through Intestinal Barrier Protection and Gut Microbiota Modulation.” Antioxidants 12, no. 5: 1122. 37237988 10.3390/antiox12051122 PMC10215682

[cbdd70371-bib-0010] Guo, K. , X. He , J. Zou , and Y. Tang . 2025. “Commentary: Pingwei Powder Alleviates High‐Fat Diet‐Induced Colonic Inflammation by Modulating Microbial Metabolites SCFAs.” Frontiers in Cellular and Infection Microbiology 15: 1709791. 10.3389/fcimb.2025.1709791. 41189706 PMC12580174

[cbdd70371-bib-0011] Harrison, S. A. , P. Bedossa , C. D. Guy , et al. 2024. “A Phase 3, Randomized, Controlled Trial of Resmetirom in NASH With Liver Fibrosis.” New England Journal of Medicine 390: 497–509. 38324483 10.1056/NEJMoa2309000

[cbdd70371-bib-0012] Harrison, S. A. , T. Rolph , M. Knott , and J. Dubourg . 2024. “FGF21 Agonists: An Emerging Therapeutic for Metabolic Dysfunction‐Associated Steatohepatitis and Beyond.” Journal of Hepatology 81, no. 3: 562–576. 38710230 10.1016/j.jhep.2024.04.034

[cbdd70371-bib-0013] He, J. , Y. Yang , F. Zhang , et al. 2022. “Effects of Poria Cocos Extract on Metabolic Dysfunction‐Associated Fatty Liver Disease via the FXR/PPARα‐SREBPs Pathway.” Frontiers in Pharmacology 13: 1007274. 10.3389/fphar.2022.1007274. 36278226 PMC9581278

[cbdd70371-bib-0014] He, Y. , Y. Chen , S. Qian , et al. 2025. “Immunopathogenic Mechanisms and Immunoregulatory Therapies in MASLD.” Cellular and Molecular Immunology 22, no. 10: 1159–1177. 40494889 10.1038/s41423-025-01307-5 PMC12480724

[cbdd70371-bib-0015] Jiang, S. , C. Liang , X. Wan , et al. 2024. “Integrative Analysis Reveals the Anti‐Obesity Roles of Poria Cocos Polysaccharides Through Beneficial Effects on Gut Microbiota.” Journal of Functional Foods 119: 106308.

[cbdd70371-bib-0016] Kong, C.‐Y. , Z.‐M. Li , B. Han , et al. 2026. “Modulation of Gut Microbiota by Tangerine Peel Polysaccharide: A Promising Strategy for Obesity Management via Acetic Acid Production.” Food Research International 229: 118460. 41763783 10.1016/j.foodres.2026.118460

[cbdd70371-bib-0017] Krahmer, N. , T. C. Walther , and R. V. Farese Jr. 2025. “The Pathogenesis of Hepatic Steatosis in MASLD: A Lipid Droplet Perspective.” Journal of Clinical Investigation 135, no. 18: e198334. 10.1172/jci198334. 40955666 PMC12435828

[cbdd70371-bib-0018] Lee, G. H. , C. Peng , S. A. Park , et al. 2020. “Citrus Peel Extract Ameliorates High‐Fat Diet‐Induced NAFLD via Activation of AMPK Signaling.” Nutrients 12, no. 3: 673. 32121602 10.3390/nu12030673 PMC7146518

[cbdd70371-bib-0019] Li, Y. S. , C. Ni , J. Wang , et al. 2015. “Lecture 23: Discussion on Medical Cases of Fatty Liver.” Bulletin of Chinese Medicine 14, no. 5: 37 (in Chinese).

[cbdd70371-bib-0020] Li, Z. , J. Xu , P. Zheng , et al. 2015. “Hawthorn Leaf Flavonoids Alleviate Nonalcoholic Fatty Liver Disease by Enhancing the Adiponectin/AMPK Pathway.” International Journal of Clinical and Experimental Medicine 8, no. 10: 17295. 26770322 PMC4694222

[cbdd70371-bib-0021] Liu, D. , C. C. Wong , Y. Zhou , et al. 2021. “Squalene Epoxidase Induces Nonalcoholic Steatohepatitis via Binding to Carbonic Anhydrase III and Is a Therapeutic Target.” Gastroenterology 160, no. 7: 2467–2482. 10.1053/j.gastro.2021.02.051. 33647280

[cbdd70371-bib-0022] Luo, Y. , Y. Zheng , J. Qin , et al. 2026. “Targeting the Cytokine‐Epigenetic Axis: A New Paradigm and Prospects for Disease Treatment.” European Cytokine Network 37, no. 2: 97–119. 42421500 10.32604/ecn.2026.082885

[cbdd70371-bib-0023] Miao, J. , J. Lin , J. Dong , et al. 2024. “Discovery and Evaluation of Novel SHIP‐1 Inhibitors.” Bioorganic & Medicinal Chemistry 114: 117965. 10.1016/j.bmc.2024.117965. 39454561 PMC11551725

[cbdd70371-bib-0024] Miao, R. , Y. Zhang , Y. Zhang , X. Fang , R. Yin , and J. Tian . 2025. “Jiangtang Tiaozhi Formula Ameliorates MASLD by Regulating Liver ABCD2/PEX2/ATGL Axis‐Mediated Fatty Acid Metabolic Reprogramming.” Phytomedicine 145: 157032. 40674914 10.1016/j.phymed.2025.157032

[cbdd70371-bib-0025] Nguyen, V. , E. G. Taine , D. Meng , T. Cui , and W. Tan . 2024. “Chlorogenic Acid: A Systematic Review on the Biological Functions, Mechanistic Actions, and Therapeutic Potentials.” Nutrients 16, no. 7: 924. 38612964 10.3390/nu16070924 PMC11013850

[cbdd70371-bib-0026] Ozkan‐Nikitaras, T. , D. J. Grzesik , L. E. Romano , J. Chapple , P. J. King , and C. C. Shoulders . 2024. “N‐SREBP2 Provides a Mechanism for Dynamic Control of Cellular Cholesterol Homeostasis.” Cells 13, no. 15: 1255. 39120286 10.3390/cells13151255 PMC11311687

[cbdd70371-bib-0027] Pengnet, S. , P. Sumarithum , N. Phongnu , et al. 2022. “Naringin Attenuates Fructose‐Induced NAFLD Progression in Rats Through Reducing Endogenous Triglyceride Synthesis and Activating the Nrf2/HO‐1 Pathway.” Frontiers in Pharmacology 13: 1049818. 36588703 10.3389/fphar.2022.1049818 PMC9797507

[cbdd70371-bib-0028] Sanyal, A. J. , P. N. Newsome , I. Kliers , et al. 2025. “Phase 3 Trial of Semaglutide in Metabolic Dysfunction‐Associated Steatohepatitis.” New England Journal of Medicine 392, no. 21: 2089–2099. 40305708 10.1056/NEJMoa2413258

[cbdd70371-bib-0029] Sheikh, M. Y. , M. F. Younus , A. Shergill , and M. N. Hasan . 2025. “Diet and Lifestyle Interventions in Metabolic Dysfunction‐Associated Fatty Liver Disease: A Comprehensive Review.” International Journal of Molecular Sciences 26, no. 19: 9625. 41096891 10.3390/ijms26199625 PMC12524441

[cbdd70371-bib-0030] Sun, W. , J. Jia , G. Liu , et al. 2025. “Polysaccharides Extracted From Old Stalks of *Asparagus officinalis* L. Improve Nonalcoholic Fatty Liver by Increasing the Gut Butyric Acid Content and Improving Gut Barrier Function.” Journal of Agricultural and Food Chemistry 73, no. 11: 6632–6645. 40042965 10.1021/acs.jafc.4c07078

[cbdd70371-bib-0031] Sun, W. , N. Xue , and Q. Zhang . 2025. “Integrated Multi‐Omics Analysis Reveals the Mechanisms of Naringin in Ameliorating High‐Fat Diet‐Induced Metabolic Dysfunction‐Associated Steatotic Liver Disease.” Frontiers in Nutrition 12: 1694191. 41195064 10.3389/fnut.2025.1694191 PMC12583168

[cbdd70371-bib-0032] Tao, S.‐H. , Y.‐Q. Lei , Y.‐M. Tan , Y.‐B. Yang , and W.‐N. Xie . 2024. “Chinese Herbal Formula in the Treatment of Metabolic Dysfunction‐Associated Steatotic Liver Disease: Current Evidence and Practice.” Frontiers in Medicine 11: 1476419. 39440040 10.3389/fmed.2024.1476419 PMC11493624

[cbdd70371-bib-0033] Venkatesan, N. , L. C. Doskey , and H. Malhi . 2023. “The Role of Endoplasmic Reticulum in Lipotoxicity During Metabolic Dysfunction–Associated Steatotic Liver Disease (MASLD) Pathogenesis.” American Journal of Pathology 193, no. 12: 1887–1899. 37689385 10.1016/j.ajpath.2023.08.007 PMC10699131

[cbdd70371-bib-0034] Wu, Y. , Z. Chen , Y. Hu , et al. 2026. “SUMOylation Regulates Tumorigenesis and Progression: Molecular Mechanisms and Therapeutic Applications.” Interdisciplinary Medicine 4, no. 3: e70120. 10.1002/inmd.70120.

[cbdd70371-bib-0035] Yu, Y. Y. , M. Feng , Y. Chen , et al. 2025. “Asprosin‐FABP5 Interaction Modulates Mitochondrial Fatty Acid Oxidation Through PPARα Contributing to MASLD Development.” Advanced Science (Weinheim, Baden‐Wurttemberg, Germany) 12, no. 21: 2415846. 40231957 10.1002/advs.202415846 PMC12140288

[cbdd70371-bib-0036] Zhao, N. , M. Dang , Y. Sun , et al. 2025. “Integrated Chemical Composition, Transcriptomics, and Network Pharmacology to Reveal the Mechanism of Jia‐Wei‐Si‐Miao‐Yong‐An Decoction in ACS Model Rats.” Phytomedicine 145: 157027. 40645073 10.1016/j.phymed.2025.157027

[cbdd70371-bib-0037] Zheng, N. , H. Wang , W. Zhu , Y. Li , and H. Li . 2024. “Astragalus Polysaccharide Attenuates Nonalcoholic Fatty Liver Disease Through THDCA in High‐Fat Diet‐Fed Mice.” Journal of Ethnopharmacology 320: 117401. 37967775 10.1016/j.jep.2023.117401

[cbdd70371-bib-0038] Zhong, J. , X. He , X. Gao , et al. 2023. “Hyodeoxycholic Acid Ameliorates Nonalcoholic Fatty Liver Disease by Inhibiting RAN‐Mediated PPARα Nucleus‐Cytoplasm Shuttling.” Nature Communications 14, no. 1: 5451. 10.1038/s41467-023-41061-8 PMC10482907 37673856

[cbdd70371-bib-0039] Zhou, X. , Y. Zhuang , X. Liu , et al. 2023. “Study on Tumour Cell‐Derived Hybrid Exosomes as Dasatinib Nanocarriers for Pancreatic Cancer Therapy.” Artificial Cells, Nanomedicine, and Biotechnology 51, no. 1: 532–546. 10.1080/21691401.2023.2264358. 37948136

[cbdd70371-bib-0040] Ziółkiewicz, A. , P. Niziński , J. Soja , et al. 2024. “Potential of Chlorogenic Acid in the Management of Metabolic Dysfunction‐Associated Steatotic Liver Disease (MASLD): Animal Studies and Clinical Trials—A Narrative Review.” Metabolites 14, no. 6: 346. 38921480 10.3390/metabo14060346 PMC11205996

[cbdd70371-bib-0041] Zou, J. , Y. Dai , Y. Yu , Y. Wu , Q. Xiang , and R. Yu . 2026. “Integrated Analysis of Network Pharmacology and Multi‐Omics Reveals the Mechanisms of Zuogui Jiangtang Qinggan Formula Ameliorates MASLD via Fatty Acid Metabolic Reprogramming.” Phytomedicine 155: 158128. 41962267 10.1016/j.phymed.2026.158128

