# Supplementary material for: Jiuwei Xiaozhi Decoction Alleviates High‐Fat Diet‐Induced MASLD by Suppressing Hepatic SREBP2‐Driven Cholesterogenesis and Restoring PPARα‐Mediated Fatty Acid Oxidation
Source: Chem Biol Drug Des. 2026 Jul 28;108(2):e70371. doi: 10.1111/cbdd.70371 (PMC13416012; doi:10.1111/cbdd.70371)
Supplement: Supplementary file 1 — Figure S1: Total ion chromatograms of the blank sample. (A) TIC of the blank sample acquired in positive ion mode. (B) TIC of the blank sample acquired in negative ion mode. The blank sample was prepared and analyzed under the same UHPLC‐HRMS/MS conditions as JWXZ to assess background signals and potential carry‐over interference. Table S1: Detailed composition of the diets. Table S2: Complete chemical constituents identified in JWXZ by UHPLC‐HRMS/MS. Table S3: Complete molecular docking results. Table S4: Antibodies used for Western blotting and immunofluorescence staining. [file CBDD-108-e70371-s001.docx]

Supplementary Material

# Supplementary Figures

**Supplementary Figure 1.** Total ion chromatograms of the blank sample. (A) TIC of the blank sample acquired in positive ion mode. (B) TIC of the blank sample acquired in negative ion mode. The blank sample was prepared and analyzed under the same UHPLC-HRMS/MS conditions as JWXZ to assess background signals and potential carry-over interference.

# Supplementary Tables

# **Supplementary Table S1.** Detailed composition of the diets

|  | **NC group** | | **HFD group** | |
| --- | --- | --- | --- | --- |
|  | **g** | **kcal** | **g** | **kcal** |
| Casein, 80 Mesh | 200 | 800 | 200 | 800 |
| L-Cystine | 3 | 12 | 3 | 12 |
| Corn Starch | 397 | 1590 | 0 | 0 |
| Maltodextrin 10 | 132 | 528 | 125 | 500 |
| Sucrose | 100 | 400 | 68.8 | 275.2 |
| Cellulose, BW200 | 50 | 0 | 50 | 0 |
| Soybean Oil | 70 | 630 | 25 | 225 |
| t-Butylhydroquinone | 0.014 | 0 | 0 | 0 |
| Lard | 0 | 0 | 245 | 2205 |
| Mineral Mix, S10026 | 35 | 0 | 10 | 0 |
| DiCalcium Phosphate | 0 | 0 | 13 | 0 |
| Calcium Carbonate | 0 | 0 | 5.5 | 0 |
| Potassium Citrate, 1 H2O | 0 | 0 | 16.5 | 0 |
| Vitamin Mix, V10001 | 10 | 40 | 10 | 40 |
| Choline Bitartrate | 2.5 | 0 | 2 | 0 |
| FD&C Blue Dye #1 | 0 | 0 | 0.05 | 0 |
| Total | 999.514 | 4000 | 773.85 | 4057.2 |

**Supplementary Table S2** Complete chemical constituents identified in JWXZ by UHPLC-HRMS/MS.

| No. | Compound | Molecular formula | Mass error (ppm) | Molecular weight | RT (min) | Match score | Peak area | Relative content (%) |
| --- | --- | --- | --- | --- | --- | --- | --- | --- |
| 1 | Citric acid | C6H8O7 | -0.77 | 192.02685 | 1.877 | 91.8 | 26378531502 | 14.264 |
| 2 | Naringin | C27H32O14 | -0.83 | 580.17873 | 23.587 | 90 | 20999480365 | 11.356 |
| 3 | Sinapine | C16H23NO5 | 0.45 | 309.15776 | 20.850 | 89.1 | 14817316952 | 8.013 |
| 4 | Gluconic acid | C6H12O7 | -1.4 | 196.05803 | 1.542 | 95.8 | 9885665506 | 5.346 |
| 5 | Choline | C5H13NO | 1.89 | 103.09991 | 1.516 | 95.3 | 8917508632 | 4.822 |
| 6 | Naringenin chalcone | C15H12O5 | 0.09 | 272.06850 | 23.582 | 90.1 | 8669441585 | 4.688 |
| 7 | DL-Arginine | C6H14N4O2 | 1.71 | 174.11197 | 1.461 | 94.6 | 7737619170 | 4.184 |
| 8 | 2-Pyrrolidinecarboxylic acid | C5H9NO2 | 1.63 | 115.06352 | 1.631 | 84.3 | 7729395596 | 4.180 |
| 9 | L-(-)-Malic acid | C4H6O5 | -1.56 | 134.02131 | 1.665 | 97.7 | 5422106168 | 2.932 |
| 10 | Quinic acid | C7H12O6 | -0.8 | 192.06323 | 1.615 | 88.9 | 4605640259 | 2.491 |
| 11 | Chlorogenic acid | C16H18O9 | -0.77 | 354.09481 | 20.237 | 93.1 | 4257595863 | 2.302 |
| 12 | Sucrose | C12H22O11 | -0.77 | 342.11595 | 1.642 | 93.8 | 4136305792 | 2.237 |
| 13 | Corchorifatty acid F | C18H32O5 | -0.98 | 328.22465 | 28.740 | 88.9 | 4019875841 | 2.174 |
| 14 | 2-Furoic acid | C5H4O3 | -2.26 | 112.01579 | 1.873 | 93.5 | 2962067527 | 1.602 |
| 15 | Cryptochlorogenic acid | C16H18O9 | -0.92 | 354.09476 | 19.154 | 92 | 2494328701 | 1.349 |
| 16 | Trigonelline | C7H7NO2 | 1.51 | 137.04789 | 1.625 | 86.8 | 2288951140 | 1.238 |
| 17 | Rhoifolin | C27H30O14 | 0.3 | 578.16373 | 23.544 | 82.9 | 2288472556 | 1.238 |
| 18 | Isoquercitrin | C21H20O12 | 0.11 | 464.09553 | 22.861 | 93.6 | 2122302189 | 1.148 |
| 19 | δ-Gluconic acid δ-lactone | C6H10O6 | -1 | 178.04756 | 2.841 | 77.1 | 1760492152 | 0.952 |
| 20 | Mannitol | C6H14O6 | 0.05 | 182.07905 | 1.537 | 87.4 | 1721822781 | 0.931 |
| 21 | Adenosine | C10H13N5O4 | 1.4 | 267.09713 | 11.955 | 89.2 | 1716571875 | 0.928 |
| 22 | Calycosin-7-O-β-D-glucoside | C22H22O10 | 0.61 | 446.12157 | 22.491 | 87.2 | 1509472740 | 0.816 |
| 23 | Azelaic acid | C9H16O4 | -1.21 | 188.10463 | 24.491 | 76.4 | 1297191008 | 0.701 |
| 24 | Vicenin II | C27H30O15 | 0.27 | 594.15863 | 20.742 | 82.8 | 1176902299 | 0.636 |
| 25 | Isomeranzin | C15H16O4 | 0.6 | 260.10502 | 32.359 | 89.9 | 1110858820 | 0.601 |
| 26 | Calycosin | C16H12O5 | -0.04 | 284.06846 | 27.015 | 91.2 | 1068275779 | 0.578 |
| 27 | Guanosine-3',5'-cyclic monophosphate | C10H12N5O7P | 0.22 | 345.04751 | 9.322 | 81.1 | 1044101261 | 0.565 |
| 28 | Morin | C15H10O7 | 0.9 | 302.04292 | 22.870 | 87.8 | 1038349936 | 0.561 |
| 29 | Caffeic acid | C9H8O4 | -0.86 | 180.04210 | 20.969 | 88.5 | 999760617.6 | 0.541 |
| 30 | Asparagine | C4H8N2O3 | -0.34 | 132.05345 | 1.463 | 90.6 | 899551023.7 | 0.486 |
| 31 | 4-Oxoproline | C5H7NO3 | 0.25 | 129.04263 | 4.037 | 56.2 | 898219246.3 | 0.486 |
| 32 | Kaempferol | C15H10O6 | 0.35 | 286.04784 | 23.636 | 88.8 | 884425836.3 | 0.478 |
| 33 | Formononetin | C16H12O4 | 0.06 | 268.07358 | 31.309 | 90.7 | 877135534.7 | 0.474 |
| 34 | Stachyose | C24H42O21 | 0.08 | 666.22191 | 5.014 | 74.6 | 860736119.7 | 0.465 |
| 35 | Rutin | C27H30O16 | -0.24 | 610.15324 | 22.392 | 92.5 | 859616247.2 | 0.465 |
| 36 | Succinic acid | C4H6O4 | -1.54 | 118.02643 | 4.810 | 94.2 | 847988200.8 | 0.459 |
| 37 | Ononin | C22H22O9 | -0.92 | 196.08556 | 25.221 | 89.6 | 817931109.4 | 0.442 |
| 38 | 3',6-Disinapoyl sucrose 3', | C34H42O19 | -1.03 | 754.23125 | 23.698 | 93.2 | 806868500.1 | 0.436 |
| 39 | L-Valine | C5H11NO2 | 1.65 | 117.07917 | 1.848 | 77.7 | 786544631.3 | 0.425 |
| 40 | 2-Isopropylmalic acid | C7H12O5 | -1.44 | 176.06822 | 19.961 | 97.8 | 766213523.9 | 0.414 |
| 41 | Astragalin | C21H20O11 | -0.47 | 448.10035 | 23.655 | 92.9 | 762469458 | 0.412 |
| 42 | Salicylic acid | C7H6O3 | -1.79 | 138.03145 | 25.159 | 85.7 | 757254376 | 0.409 |
| 43 | Isoguanosine | C10H13N5O5 | 0.27 | 283.09174 | 14.617 | 84.8 | 723954008.8 | 0.391 |
| 44 | Maleic acid | C4H4O4 | -2.2 | 116.01070 | 4.246 | 71.1 | 705079641.5 | 0.381 |
| 45 | L-Aspartic acid | C4H7NO4 | -1.29 | 133.03734 | 1.469 | 81.8 | 685504077.1 | 0.371 |
| 46 | Manninotriose | C18H32O16 | 0.4 | 504.16924 | 2.684 | 83.4 | 653105381.7 | 0.353 |
| 47 | Uridine | C9H12N2O6 | -0.68 | 244.06937 | 6.066 | 91.9 | 592947110.7 | 0.321 |
| 48 | Limonin | C26H30O8 | -0.92 | 392.18089 | 32.850 | 84.3 | 574401747.4 | 0.311 |
| 49 | α-Lapachone | C15H14O3 | 0.72 | 242.09447 | 25.497 | 72.7 | 574205332.3 | 0.311 |
| 50 | Epicatechin | C15H14O6 | -0.06 | 290.07902 | 21.141 | 93 | 560697683.8 | 0.303 |
| 51 | 5-Hydroxymethylfurfural | C6H6O3 | 1.25 | 126.03185 | 5.220 | 83.8 | 497558753.3 | 0.269 |
| 52 | N-Acetyl-DL-glutamic acid | C7H11NO5 | -0.5 | 189.06363 | 3.011 | 94.8 | 484454859.2 | 0.262 |
| 53 | Methylnissolin-3-O-glucoside | C23H26O10 | -0.34 | 462.15244 | 26.072 | 81.8 | 478470125.8 | 0.259 |
| 54 | Nicotinic acid | C6H5NO2 | 2.18 | 123.03230 | 3.025 | 82.5 | 454666619.1 | 0.246 |
| 55 | Eriocitrin | C27H32O15 | -0.19 | 596.17400 | 22.704 | 72.6 | 443436373.7 | 0.240 |
| 56 | D-(+)-Galactose | C6H12O6 | -1.13 | 180.06318 | 1.576 | 90 | 435087643.3 | 0.235 |
| 57 | p-Coumaric acid | C9H8O3 | 2.66 | 164.04778 | 5.780 | 80.6 | 409680606.3 | 0.222 |
| 58 | Protocatechualdehyde | C7H6O3 | -1.2 | 138.03153 | 19.702 | 85.4 | 403494628.7 | 0.218 |
| 59 | N-Acetyl-L-phenylalanine | C11H13NO3 | -1.5 | 207.08923 | 22.454 | 95.9 | 400514784.7 | 0.217 |
| 60 | Quercetin-3-O-beta-glucopyranosyl-6'-acetate | C23H22O13 | -0.14 | 506.10597 | 23.269 | 91.6 | 387196338.1 | 0.209 |
| 61 | Myrislignan | C21H26O6 | 0.27 | 396.15526 | 36.752 | 92.2 | 367334148.4 | 0.199 |
| 62 | 1-Deoxynojirimycin | C6H13NO4 | 1.79 | 163.08475 | 1.478 | 79.4 | 360518735 | 0.195 |
| 63 | 6-Methylquinoline | C10H9N | 2.44 | 143.07385 | 20.364 | 71.5 | 336314182.1 | 0.182 |
| 64 | Naringenin | C15H12O5 | -0.66 | 272.06830 | 28.861 | 89.7 | 335746002.2 | 0.182 |
| 65 | Apigenin | C15H10O5 | -1.06 | 270.05254 | 23.551 | 77.8 | 329788037.6 | 0.178 |
| 66 | Adenosine 3',5'-cyclic monophosphate | C10H12N5O6P | 0.88 | 329.05281 | 7.084 | 78.1 | 329291563.2 | 0.178 |
| 67 | Uracil | C4H4N2O2 | 1.72 | 112.02747 | 6.083 | 76.9 | 305343660.2 | 0.165 |
| 68 | Esculetin | C9H6O4 | -1.19 | 178.02640 | 20.901 | 85.3 | 285551295.9 | 0.154 |
| 69 | L-Pyroglutamic acid | C5H7NO3 | 2.69 | 129.04294 | 4.797 | 85.5 | 282211647.1 | 0.153 |
| 70 | 4-Ethynylaniline | C8H7N | 1.55 | 117.05803 | 19.226 | 80.2 | 281321133.9 | 0.152 |
| 71 | Glutaconic acid | C5H6O4 | -1.4 | 130.02643 | 1.902 | 93.3 | 277297208.4 | 0.150 |
| 72 | Quercetin | C15H10O7 | -0.5 | 302.04250 | 27.042 | 92.4 | 271786877.5 | 0.147 |
| 73 | Cytosine | C4H5N3O | 2.19 | 111.04350 | 3.094 | 85.1 | 260981567.9 | 0.141 |
| 74 | D-Saccharic acid | C6H10O8 | -1.24 | 210.03731 | 1.541 | 77.3 | 248202042.6 | 0.134 |
| 75 | 7-Hydroxycoumarin | C9H6O3 | 1.13 | 162.03188 | 30.416 | 80.2 | 218608349.6 | 0.118 |
| 76 | Protocatechuic acid | C7H6O4 | -0.98 | 154.02646 | 18.043 | 88.2 | 201409236.2 | 0.109 |
| 77 | Isomucronulatol 7-O-glucoside | C23H28O10 | -0.06 | 464.16822 | 26.442 | 89 | 196979809.1 | 0.107 |
| 78 | Isochlorogenic acid C | C25H24O12 | -0.69 | 516.12642 | 24.067 | 88.8 | 196785909 | 0.106 |
| 79 | Kaempferol-3-O-rutinoside | C27H30O15 | -0.05 | 594.15844 | 23.161 | 80.6 | 188587655.7 | 0.102 |
| 80 | Obacunone | C26H30O7 | 0.85 | 454.19954 | 36.474 | 79.4 | 185996967.7 | 0.101 |
| 81 | 4-Guanidinobutyric acid | C5H11N3O2 | 1.71 | 145.08537 | 2.963 | 75.4 | 185444200.1 | 0.100 |
| 82 | Acetylarginine | C8H16N4O3 | 2.33 | 216.12274 | 3.123 | 77.6 | 183839175.3 | 0.099 |
| 83 | Dehydrocostus lactone | C15H18O2 | 1.05 | 230.13092 | 35.514 | 84 | 181144771.4 | 0.098 |
| 84 | Xanthine | C5H4N4O2 | 0.68 | 152.03353 | 5.283 | 65.2 | 164821012.3 | 0.089 |
| 85 | 6''-O-Acetylglycitin 6''-O- | C24H24O11 | 1.28 | 488.13249 | 24.972 | 83 | 158062067 | 0.085 |
| 86 | α-Linolenic acid | C18H30O2 | 1.48 | 278.22499 | 42.113 | 90.1 | 144620939.9 | 0.078 |
| 87 | Suberic acid | C8H14O4 | -1.7 | 174.08891 | 22.581 | 95.4 | 140390669.1 | 0.076 |
| 88 | Kojic acid | C6H6O4 | 2.5 | 142.02696 | 18.549 | 59.3 | 133233549.2 | 0.072 |
| 89 | Adenine | C5H5N5 | -1.07 | 135.05435 | 12.001 | 79.4 | 123465408.8 | 0.067 |
| 90 | Guanosine monophosphate (GMP) | C10H14N5O8P | 0.39 | 363.05814 | 7.026 | 91.7 | 120779689.6 | 0.065 |
| 91 | Adenosine 5'-monophosphate | C10H14N5O7P | 1.18 | 347.06349 | 3.532 | 81.9 | 117753201.8 | 0.064 |
| 92 | Procyanidin B2 | C30H26O12 | 0.87 | 578.14293 | 20.598 | 87.8 | 117467941.1 | 0.064 |
| 93 | Eriodictyol | C15H12O6 | 1.01 | 288.06368 | 22.608 | 79.3 | 116684960.8 | 0.063 |
| 94 | Sinapic acid | C11H12O5 | -0.78 | 224.06830 | 23.243 | 84.2 | 111872861.2 | 0.060 |
| 95 | Isochlorogenic acid B | C25H24O12 | -0.42 | 516.12656 | 23.266 | 88.5 | 109666876.7 | 0.059 |
| 96 | Cordycepin | C10H13N5O3 | 1.52 | 251.10222 | 13.950 | 82.8 | 108984669.5 | 0.059 |
| 97 | Trehalose-6-Phosphate | C12H23O14P | 0.05 | 422.08256 | 1.536 | 81.8 | 107395896.4 | 0.058 |
| 98 | Vitexin | C21H20O10 | -0.06 | 432.10562 | 22.560 | 81.9 | 101501181.7 | 0.055 |
| 99 | 3-Methoxybenzaldehyde | C8H8O2 | 2.82 | 136.05281 | 18.324 | 83 | 97858098.95 | 0.053 |
| 100 | Uridine monophosphate (UMP) | C9H13N2O9P | -0.83 | 324.03560 | 4.294 | 75.1 | 94731067.76 | 0.051 |
| 101 | 3,5-Dicaffeoylquinic acid | C25H24O12 | -0.3 | 516.12662 | 23.669 | 89 | 86649263.39 | 0.047 |
| 102 | Riboflavin | C17H20N4O6 | -0.33 | 376.13816 | 20.936 | 88.2 | 83707585.08 | 0.045 |
| 103 | Astragaloside IV | C41H68O14 | -0.69 | 830.46620 | 30.382 | 75.7 | 83268938.74 | 0.045 |
| 104 | Isoscopoletin | C10H8O4 | 1.29 | 192.04251 | 23.272 | 77.9 | 78030956.9 | 0.042 |
| 105 | Apigenin 7-O-(2G-rhamnosyl)gentiobioside | C33H40O19 | 1.13 | 740.21721 | 21.283 | 80.6 | 72747750.23 | 0.039 |
| 106 | Astragaloside III | C41H68O14 | -0.09 | 784.46084 | 30.669 | 92 | 70603821.41 | 0.038 |
| 107 | N-Acetyl-DL-tryptophan | C13H14N2O3 | -1.1 | 246.10017 | 23.201 | 93.2 | 69730417.88 | 0.038 |
| 108 | Jasmone | C11H16O | 1.7 | 164.12039 | 29.882 | 83.4 | 65371210.45 | 0.035 |
| 109 | Sibiricose A6 | C23H32O15 | -0.69 | 548.17374 | 21.024 | 82.8 | 64751685.22 | 0.035 |
| 110 | Gentisic acid | C7H6O4 | -1.01 | 154.02645 | 20.006 | 85.8 | 64618623.43 | 0.035 |
| 111 | 2-Methylbenzoic acid | C8H8O2 | -1.25 | 136.05226 | 20.306 | 54.9 | 60851489.29 | 0.033 |
| 112 | p-Hydroxybenzaldehyde | C7H6O2 | -0.91 | 122.03667 | 21.496 | 72 | 55910180.69 | 0.030 |
| 113 | Pinocembrin | C15H12O4 | 0.62 | 256.07372 | 26.446 | 82.9 | 55530239.75 | 0.030 |
| 114 | Hyperoside | C21H20O12 | 1.91 | 464.09636 | 19.979 | 82.5 | 55293259.39 | 0.030 |
| 115 | Shikimic acid | C7H10O5 | -2.19 | 174.05244 | 20.319 | 76.2 | 54991979.64 | 0.030 |
| 116 | Methyl hexadecanoate | C17H34O2 | -1.01 | 316.26106 | 39.730 | 79.2 | 52333525.71 | 0.028 |
| 117 | Pachymic acid | C33H52O5 | -0.56 | 528.38118 | 47.390 | 79.3 | 52160106.59 | 0.028 |
| 118 | Citropten | C11H10O4 | 0.52 | 206.05802 | 22.065 | 77.7 | 50659948.5 | 0.027 |
| 119 | Tectoridin | C22H22O11 | 0.68 | 462.11653 | 23.989 | 74.4 | 48022125.51 | 0.026 |
| 120 | N-Acetyl-L-leucine | C8H15NO3 | -0.62 | 173.10509 | 21.557 | 91.1 | 47395931.32 | 0.026 |
| 121 | Xanthosine | C10H12N4O6 | -0.49 | 284.07554 | 17.966 | 81 | 47165191.48 | 0.026 |
| 122 | Lariciresinol 4-O-glucoside | C26H34O11 | -0.11 | 522.21006 | 22.559 | 83.4 | 46378713.32 | 0.025 |
| 123 | Atractylenolide II | C15H20O2 | 0.87 | 232.14653 | 39.912 | 89.5 | 44374281.78 | 0.024 |
| 124 | Vitexin rhamnoside | C27H30O14 | 0.04 | 578.16358 | 22.247 | 81.4 | 44288423.83 | 0.024 |
| 125 | L-Phenylalanine | C9H11NO2 | -2.04 | 165.07864 | 22.452 | 86.4 | 43611395.29 | 0.024 |
| 126 | Grosvenorine | C33H40O19 | 0.73 | 740.21692 | 21.961 | 84.5 | 42635047.7 | 0.023 |
| 127 | Diosmin | C28H32O15 | 0.66 | 608.17452 | 23.796 | 75.9 | 40700414.25 | 0.022 |
| 128 | Abscisic acid | C15H20O4 | -1.3 | 264.13582 | 25.604 | 83.7 | 40563695.99 | 0.022 |
| 129 | Arglabin | C15H18O3 | 1.14 | 246.12588 | 29.421 | 76.4 | 39976566.24 | 0.022 |
| 130 | Mulberroside A | C26H32O14 | -0.09 | 568.17916 | 19.753 | 82.8 | 39198270.08 | 0.021 |
| 131 | Inosine | C10H12N4O5 | -0.71 | 268.08058 | 14.305 | 80.3 | 37364251.32 | 0.020 |
| 132 | Kaempferol-7-O-β-D-glucopyranoside | C21H20O11 | 1.73 | 448.10134 | 20.434 | 79.7 | 34749765.18 | 0.019 |
| 133 | Leucylproline | C11H20N2O3 | 1.45 | 228.14772 | 18.110 | 76.8 | 32483184.67 | 0.018 |
| 134 | Parthenolide | C15H20O3 | 0.53 | 248.14137 | 26.608 | 86.2 | 31778584.21 | 0.017 |
| 135 | 1,3-Dicaffeoylquinic acid | C25H24O12 | -0.34 | 516.12660 | 21.312 | 82.8 | 31380276.73 | 0.017 |
| 136 | Soyasaponin I | C48H78O18 | -0.29 | 942.51854 | 32.405 | 85.9 | 30608692.05 | 0.017 |
| 137 | Scopoletin | C10H8O4 | 1.8 | 192.04260 | 20.281 | 71.5 | 30058804.13 | 0.016 |
| 138 | 7-Methoxycoumarin | C10H8O3 | 1.51 | 176.04761 | 25.510 | 74.9 | 29038475.17 | 0.016 |
| 139 | 2-Hydroxy-4-methoxybenzaldehyde | C8H8O3 | 1.74 | 152.04761 | 22.610 | 80.9 | 28192212.96 | 0.015 |
| 140 | Coumarin | C9H6O2 | 0.29 | 146.03682 | 21.337 | 73 | 28145096.71 | 0.015 |
| 141 | 2-Hydroxycaproic acid | C6H12O3 | -1.78 | 132.07841 | 21.320 | 90.2 | 27919648.28 | 0.015 |
| 142 | Artemisinic acid | C15H22O2 | 1.51 | 252.17280 | 21.740 | 82.3 | 27473502.98 | 0.015 |
| 143 | 4-Nitrophenol | C6H5NO3 | -1.52 | 139.02673 | 25.334 | 85.9 | 26180272.94 | 0.014 |
| 144 | Indole-3-carboxaldehyde | C9H7NO | 1.6 | 145.05300 | 19.622 | 82.2 | 25013336.25 | 0.014 |
| 145 | Atractylenolide I | C15H18O2 | 1.05 | 230.13092 | 42.924 | 84.2 | 24646579.63 | 0.013 |
| 146 | Tinnevellin glucoside | C20H24O9 | -0.27 | 408.14192 | 25.474 | 72.5 | 24202926.37 | 0.013 |
| 147 | Curcumol | C15H24O2 | 0.86 | 236.17783 | 29.027 | 75.6 | 22948025.39 | 0.012 |
| 148 | Curdione | C15H24O2 | 0.64 | 236.17778 | 23.366 | 75.1 | 22640326.54 | 0.012 |
| 149 | Raffinose | C18H32O16 | 1.51 | 550.17449 | 3.419 | 81.2 | 22484653.52 | 0.012 |
| 150 | Deoxyandrographolide | C20H30O4 | -6.68 | 334.21218 | 37.612 | 73.3 | 21478672.36 | 0.012 |
| 151 | α-Cyperone | C15H22O | -0.09 | 236.17787 | 30.937 | 78.7 | 20845803.11 | 0.011 |
| 152 | Hydroxygenkwanin | C16H12O6 | -0.17 | 300.06334 | 29.830 | 81 | 20676118.52 | 0.011 |
| 153 | 3,5-Dimethoxy-4-hydroxybenzaldehyde | C9H10O4 | 1.68 | 182.05821 | 23.010 | 81.5 | 20448372.5 | 0.011 |
| 154 | Dehydrotumulosic acid | C31H48O4 | -0.55 | 484.35499 | 42.508 | 75.8 | 19568332.75 | 0.011 |
| 155 | Bergaptol | C11H6O4 | 1.16 | 202.02684 | 29.502 | 70.4 | 19214044.74 | 0.010 |
| 156 | Rosarin | C20H28O10 | -0.76 | 428.16792 | 24.152 | 71.9 | 19206460.28 | 0.010 |
| 157 | N-Acetyl-DL-norvaline | C7H13NO3 | -1.77 | 159.08926 | 19.187 | 82.8 | 18787352.98 | 0.010 |
| 158 | Ligustilide | C12H14O2 | 1.14 | 190.09960 | 24.516 | 82.6 | 18268524.26 | 0.010 |
| 159 | Parishin E | C19H24O13 | 0.06 | 460.12172 | 19.548 | 78.3 | 16907622.26 | 0.009 |
| 160 | Genistein | C15H10O5 | -1.06 | 270.05254 | 21.282 | 79.3 | 16678438.12 | 0.009 |
| 161 | α-Asarone | C12H16O3 | 1.38 | 208.11023 | 43.029 | 71.9 | 16640740.16 | 0.009 |
| 162 | Ferulaldehyde | C10H10O3 | 1.31 | 178.06323 | 36.769 | 76.6 | 16140338.64 | 0.009 |
| 163 | Germacrone | C15H22O | 1.2 | 218.16733 | 25.165 | 78.2 | 16035622.22 | 0.009 |
| 164 | Orientin | C21H20O11 | 0.01 | 448.10057 | 21.892 | 79 | 15800023.04 | 0.009 |
| 165 | Benzoic acid | C7H6O2 | 1.22 | 122.03693 | 17.900 | 79.3 | 14599135 | 0.008 |
| 166 | 5-Aminolevulinic acid | C5H9NO3 | -1.41 | 131.05806 | 5.303 | 79.9 | 14561007.99 | 0.008 |
| 167 | 5-Hydroxy-1-tetralone | C10H10O2 | 1.36 | 162.06830 | 35.501 | 80.4 | 14409314.38 | 0.008 |
| 168 | Linoleoyl Ethanolamide | C20H37NO2 | 1.41 | 323.28288 | 45.336 | 83.6 | 14180407.01 | 0.008 |
| 169 | Isoalantolactone | C15H20O2 | 0.87 | 232.14653 | 34.921 | 84.7 | 12695817.01 | 0.007 |
| 170 | Formononetin | C16H12O4 | -0.9 | 268.07332 | 27.688 | 50.5 | 12328513.06 | 0.007 |
| 171 | Nuciferine | C19H21NO2 | 1.08 | 295.15755 | 25.368 | 73.4 | 12077579.95 | 0.007 |
| 172 | Senkyunolide A | C12H16O2 | 1.61 | 192.11534 | 29.883 | 75.9 | 10121435.08 | 0.005 |
| 173 | Osthole | C15H16O3 | 1.49 | 244.11031 | 39.097 | 80.3 | 9739365.236 | 0.005 |
| 174 | Clareolide | C16H26O2 | 1.42 | 250.19364 | 37.644 | 75 | 9049974.767 | 0.005 |
| 175 | Hexadecanedioic acid | C16H30O4 | -0.57 | 286.21425 | 39.519 | 83.4 | 4190647.183 | 0.002 |
| 176 | Suberosin | C15H16O3 | 0.06 | 212.04794 | 29.511 | 83.7 | 1147286.266 | 0.001 |

**Note.** RT, retention time. Relative content was calculated based on peak area and is expressed as a percentage of the total area of the identified constituents. The table was sorted by relative content in descending order.

**Supplementary Table S3.** Complete molecular docking results.

| **No.** | **Target** | **Structure ID** | **Compound** | **Binding affinity (kcal/mol)** |
| --- | --- | --- | --- | --- |
| 1 | EGFR | PDB: 6LUD | Chlorogenic acid | -7.2 |
| 2 | EGFR | PDB: 6LUD | Sinapine | -5.6 |
| 3 | EGFR | PDB: 6LUD | Trigonelline | -4.5 |
| 4 | HMGCR | PDB: 2R4F | Calycosin | -7.5 |
| 5 | HMGCR | PDB: 2R4F | Chlorogenic acid | -7.1 |
| 6 | HMGCR | PDB: 2R4F | Naringenin chalcone | -6.3 |
| 7 | HMGCR | PDB: 2R4F | Naringin | -8.4 |
| 8 | HMGCR | PDB: 2R4F | Rhoifolin | -8.0 |
| 9 | PPARα | PDB: 6KAX | Calycosin | -8.1 |
| 10 | PPARα | PDB: 6KAX | Calycosin-7-O-β-D-glucoside | -8.6 |
| 11 | PPARα | PDB: 6KAX | Isoquercitrin | -8.0 |
| 12 | PPARα | PDB: 6KAX | Naringin | -7.5 |
| 13 | PPARα | PDB: 6KAX | Rhoifolin | -8.7 |
| 14 | SQLE | PDB: 6C6N | Calycosin | -8.6 |
| 15 | SQLE | PDB: 6C6N | Chlorogenic acid | -9.4 |
| 16 | SQLE | PDB: 6C6N | Naringenin chalcone | -9.0 |
| 17 | SREBP2 | UniProt: Q12772 | Calycosin | -6.7 |
| 18 | SREBP2 | UniProt: Q12772 | Chlorogenic acid | -6.0 |
| 19 | SREBP2 | UniProt: Q12772 | Cryptochlorogenic acid | -6.0 |
| 20 | SREBP2 | UniProt: Q12772 | Isoquercitrin | -6.8 |
| 21 | SREBP2 | UniProt: Q12772 | Naringenin chalcone | -6.2 |
| 22 | SREBP2 | UniProt: Q12772 | Naringin | -6.7 |
| 23 | SREBP2 | UniProt: Q12772 | Rhoifolin | -7.4 |
| 24 | SREBP2 | UniProt: Q12772 | Sinapine | -5.2 |

**Note.** Binding affinities were calculated using AutoDock Vina. More negative values indicate stronger predicted binding affinity. SREBP2 was modeled using the UniProt/AlphaFold structure. JWXZ, Jiuwei Xiaozhi Decoction.

**Supplementary Table S4** Antibodies used for Western blotting and immunofluorescence staining

| **Target / antibody** | **Host** | **Manufacturer** | **Catalog No.** | **Dilution** | **Application / normalization** |
| --- | --- | --- | --- | --- | --- |
| AKT | Rabbit | Proteintech | 10176-2-AP | 1:1000 | WB; total AKT |
| p-AKT | Mouse | Proteintech | 66444-1-Ig | 1:1000 | WB; normalized to AKT |
| PI3K | Rabbit | Affinity | AF6242 | 1:500 | WB; total PI3K |
| p-PI3K | Rabbit | Affinity | AF3242 | 1:500 | WB; normalized to PI3K |
| EGFR | Rabbit | Proteintech | 18986-1-AP | 1:1000 | WB; total EGFR |
| p-EGFR | Rabbit | Affinity | AF3045 | 1:500 | WB; normalized to EGFR |
| HMGCR | Rabbit | Proteintech | 13533-1-AP | 1:1000 | WB; normalized to GAPDH |
| SQLE | Rabbit | Proteintech | 12544-1-AP | 1:1000 | WB; normalized to GAPDH |
| PCSK9 | Rabbit | Affinity | DF12687 | 1:500 | WB; normalized to GAPDH |
| CPT1A | Rabbit | Proteintech | 15184-1-AP | 1:1000 | WB; normalized to GAPDH |
| SREBP2 | Rabbit | Proteintech | 28212-1-AP | 1:1000 | WB/IF; pro-SREBP2 normalized to GAPDH; N-SREBP2 normalized to Lamin B1 |
| PPARα | Mouse | Proteintech | 66826-1-Ig | 1:1000 | WB/IF; N-PPARα normalized to Lamin B1; C-PPARα normalized to GAPDH |
| Lamin B1 | Rabbit | Proteintech | 12987-1-AP | 1:1000 | Nuclear loading control |
| GAPDH | Mouse | Proteintech | 60004-1-Ig | 1:2000 | Total/cytoplasmic loading control |
| Goat anti-rabbit IgG-HRP | Goat | Proteintech | RGAR001 | 1:4000 | WB secondary antibody |
| Goat anti-mouse IgG-HRP | Goat | Proteintech | RGAM001 | 1:4000 | WB secondary antibody |
| CoraLite594-conjugated Goat Anti-Rabbit IgG(H+L) | Goat | Proteintech | SA00013-4 | 1:500 | IF secondary antibody for rabbit primary antibodies, including SREBP2 |
| CoraLite488-conjugated Goat Anti-Mouse IgG(H+L) | Goat | Proteintech | SA00013-1 | 1:500 | IF secondary antibody for mouse primary antibodies, including PPARα |
